# In-Depth Analysis Reveals Production of Circular RNAs from Non-Coding Sequences

**DOI:** 10.3390/cells9081806

**Published:** 2020-07-30

**Authors:** Annie Robic, Julie Demars, Christa Kühn

**Affiliations:** 1 GenPhySE, Université de Toulouse, INRAE, ENVT, 31326 Castanet Tolosan, France; Julie.demars@inrae.fr; 2 Institute of Genome Biology, Leibniz Institute for Farm Animal Biology (FBN), 18196 Dummerstorf, Germany; kuehn@fbn-dummerstorf.de; 3 Faculty of Agricultural and Environmental Sciences, University of Rostock, 18059 Rostock, Germany

**Keywords:** intron circle, intronic circRNA, exonic circRNA, intron, non-coding, intronic lariat, sisRNA, circular junction, sub-exonic circRNA, pig testis

## Abstract

The sequencing of total RNA depleted for ribosomal sequences remains the method of choice for the study of circRNAs. Our objective was to characterize non-canonical circRNAs, namely not originating from back splicing and circRNA produced by non-coding genes. To this end, we analyzed a dataset from porcine testis known to contain about 100 intron-derived circRNAs. Labelling reads containing a circular junction and originating from back splicing provided information on the very small contribution of long non-coding genes to the production of canonical circRNAs. Analyses of the other reads revealed two origins for non-canonical circRNAs: (1) Intronic sequences for lariat-derived intronic circRNAs and intron circles, (2) Mono-exonic genes (mostly non-coding) for either a new type of circRNA (including only part of the exon: sub-exonic circRNAs) or, even more rarely, mono-exonic canonical circRNAs. The most complex set of sub-exonic circRNAs was produced by *RNase_MRP* (ribozyme RNA). We specifically investigated the intronic circRNA of *ATXN2L*, which is probably an independently transcribed sisRNA (stable intronic sequence RNA). We may be witnessing the emergence of a new non-coding gene in the porcine genome. Our results are evidence that most non-canonical circRNAs originate from non-coding sequences.

## 1. Introduction

Although the discovery of RNA molecules with a circular configuration dates back four decades, for many years they were dismissed as being too few or as resulting from splicing errors [1]. From 2012 on, advances in high throughput sequencing revealed the presence of circular RNAs (circRNAs) in mammalian cells and in different cell lines [2]. In the meantime, evidence has accumulated that circRNAs are evolutionarily conserved and their expression levels vary with the tissue and with the developmental stage, suggesting that circRNAs have regulatory functions [3,4]. Circular RNA are probably a natural byproduct of the splicing process in all eukaryotes [5]. Splicing is the mechanism by which nascent precursor messenger RNA (pre-mRNA) is edited into mature mRNA and which is mediated by a protein–RNA complex known as the spliceosome. Typical human genes have eight introns, and each intron causes the de novo assembly of a spliceosome; multiple spliceosomes are necessary to remove introns from the pre-mRNA body. During the splicing of a pre-mRNA, introns are spliced in the form of a lariat intronic RNA and exons are joined together. The biogenesis of the two types of circRNA described so far is related to these two aspects of pre-mRNA splicing [3].

Ninety-nine percent of splicing events involve a characteristic GU at the 5′ end of the intron and an AG at the 3′ end of the intron; the two sites are termed the donor and acceptor sites, respectively [6]. In contrast to canonical splicing, which joins an upstream (5′) splice donor site and a downstream (3′) splice acceptor site, back splicing ligates a downstream splice donor site reversely with an upstream splice acceptor site, resulting in a covalently closed circRNA transcript. Back splicing is a peculiar splicing reaction that generates a class of circRNAs that can be described by identifying the two joined exons [7]. These circRNAs are called exonic circRNAs (e.g., the end of exon-4 is joined to the beginning of exon-2). Recent studies revealed that back splicing requires canonical spliceosomal machinery and can be facilitated by both complementary sequences and specific protein factors (reviewed by [3,8]). Exonic circRNAs are observed in a range of eukaryotic species, prompting speculation that back splicing is also an ancient conserved feature of the eukaryotic gene expression pathway [3].

When intronic lariats escape degradation, they can be precursors of circRNAs. Spliceosome-mediated intron excision from pre-mRNA releases a lariat molecule in which the branch-point nucleotide, usually an adenosine, links the 2′−5′ linkage to the 5′ end of the intron [9]. The spliceosome can use C or G as a branch-point nucleotide, but the lariat debranching enzyme is not efficient at hydrolyzing the 2′-5′ linkage at these residues [10]. Thus, lariats with C or G branch-points might be expected to be more stable than a standard lariat. The lariat further undergoes 3′-end trimming to form a circular sisRNA [9,11,12]. This type of intron-derived circRNA has conserved the 2′-5′ link and the sequences correspond to a lariat without a tail [9,11,12,13]. The first intronic circRNAs were identified in 2013 [13] but have been rarely investigated in mammalian tissues since then [14].

Analyzing RNA-seq data obtained after depletion of ribosomal sequences and without poly(A+) selection (Total-RNA-seq) makes it possible to identify specific circRNAs by characterizing reads that are mapped on the genome in two segments and their two segments are mapped on the genome in inverted order. Analysis of the sequence contents of these reads (including the circular junction) enabled characterization of circRNAs. Even though circular RNAs have been the topic of a number of publications (for the most recent reviews see [3,15,16,17]) only exonic circRNAs become canonical circRNAs [2,7,18]. 

The regulation of mRNA processing is of the utmost importance for differentiation, proliferation, and morphogenesis, and enables organisms to adapt to new conditions. In eukaryotes, this regulation is possible thanks to mRNA/protein complexes and in particular, to the exon junction complex [19]. Nevertheless, this description needs to be adapted for mRNAs that are produced by intron-containing genes. RNA-seq studies revealed that circRNA is expressed *in Saccharomyces cerevisiae*, in which almost no splicing involves exon skipping [20]. Mono-exonic genes have been described in eukaryotes, most are non-coding RNA and short (< 200 nt). However, some members of these small noncoding RNA families and other RNA groups (including some snoRNAs and snRNAs, as well as the 7SL, 7SK, and *RNase_MRP* RNAs) are more than 200 nucleotides in length [21]. Given the lack of knowledge on circRNAs originating from these mono-exonic genes [22], it would be interesting to try and answer this question.

In contrast to the paucity of protein coding genes, transcription and protein coding mRNAs are extraordinarily complex and contribute to a very small fraction of transcripts compared to the different varieties of non-coding RNAs (ncRNAs). What is more, the production of circRNAs by long non-coding (lnc) RNAs has not yet been analyzed. Interestingly, in mammals, the brain and testis are tissues with the highest levels of expression of both lncRNA [23] and circRNA [24,25], strongly suggesting that not only genes coding for proteins produce circRNAs. We consider the testis is the best tissue to study possible circRNAs originating from lncRNA genes since 78% of human lncRNAs reported so far are expressed in the testis [26].

This study thus focuses on non-canonical circRNAs and on the circRNA produced by non-canonical genes. The sequencing of total RNA is the method of choice for the discovery of novel classes of circRNA such as non-canonical circRNAs [3]. When we started this study, lariat-derived circRNAs were the only non-canonical circRNAs described and, in a previous study, we observed that they are rarely present in datasets, probably because of sequencing difficulties linked to the 2′-5′ links [14]. By chance, we found a porcine testicular dataset containing a large number of circRNAs and a small number of intron-derived circRNAs [14]. 

## 2. Materials and Methods

The study was conducted using a ‘wide-angle’ approach in two steps. In the first step, we labelled the exonic circRNAs and those that are likely to be exonic even if the exons involved in back splicing have not yet been described. In the second step, we examined the remaining purged list of reads with circular junctions to investigate the possible presence of non-canonical circRNAs. 

### 2.1. Datasets and Alignments

After total RNA-sequencing (total-RNA-seq: RNA-seq obtained from total RNA after ribosomal depletion) or mRNA-seq (RNA-seq of poly(A) transcripts), we obtained a dataset of stranded paired-end (PE) reads consisting of two subsets (Reads-1 and Reads-2). The datasets and their acquisition are described in detail in [14]. The datasets were obtained in two distinct sequencing runs (HiSeq2000 and HiSeq2500) but from a single library: each dataset contains a mix of 2 × 100 and 2 × 125 bp. The total-RNA-seq reads from three pubertal testes are deposited in the NCBI under accession numbers SRX5055428 (Testis-05), SRX5055429 (Testis-31), and SRX5055430 (Testis-54).

The genome assembly (Sscrofa11.1) used for all alignments corresponds to GenBank Assembly ID GCA_000003025.6 and is associated with the gene annotation v-90 (Sscrofa.11.1.90.GTF) proposed by Ensembl [27]. RNA-seq reads were mapped using the rapid splice-aware read mapper Spliced Transcripts Alignment to Sscrofa11.1 (STAR) [28]: total-RNA-seq reads were mapped with the STAR-SE option (mates of each pair were mapped independently) and reads from mRNA-seq were mapped with the STAR-PE option. We used mRNA-seq to unambiguously evaluate the expression of linear transcripts [29], and the respective reads were counted with Expectation Maximization (RSEM) software v. 1.3 [30]. 

### 2.2. Selection of Chimeric Reads Mapped in Inverted Order

Our objective was limited to selecting reads that were mapped by STAR-SE as chimeric reads with only two segments, and where both segments mapped to the same strand in inverted order in the total-RNA-seq. The methodology originally proposed by Memczak et al. in 2013 with find_circ [18] has since been adapted to currently available alignment tools [18]. As proposed by Cheng et al. (2016) [31] with DCC, we mapped reads from total-RNA-seq with STAR-SE. We used STAR with the previously proposed parameters [31] that enable the distinction of chimeric reads mapped in two segments using the minimum size for the smallest mapped segment (15 bp) (Supplementary Doc. 1.-1). STAR provides two files containing mapping information on chimeric reads: a Sam file (chimeric.out.sam) and a tabular file (chimeric.out.junction). To select reads containing a circular junction among chimeric reads, we extracted information from the tabular file (chimeric.out.junction) provided by STAR, which contains the mapping coordinates of each segment and mapping data (CIGAR). We kept reads with two fragments mapped in inverted order on the genome (Supplementary Doc. 1.-2) but when we needed a more detailed characterization of the sequence, the chimeric.out.sam file provided by STAR was used for the investigation.

### 2.3. Resources for Annotation/Labelling

The complete porcine annotation proposed by Ensembl [27] for the porcine species was analyzed (release 97) to identify exons from coding genes, from pseudogenes, from lnc transcripts, and from other non-coding (nc) genes. The annotation of porcine genome distinguishes between coding and non-coding transcripts, but never associates an nc transcript with a coding transcript in the same gene. In addition, many lncRNA transcripts have been characterized in pig but only those available in the ALDB database (domestic-Animal Long noncoding RNA DataBase, [32,33]) include the genomic coordinates of each exon. From Ensembl and ALDB, we drew up a list of exons from lncRNAs (exons-list-2). The other exons in Ensembl constitute exons-list-1. From the three double datasets (total-RNA-seq and mRNA-seq available for the three samples), we drew up a list of potential novel exons (exons-list-3) using Cufflinks and Cuffcompare [34] (see complete description in Supplementary Doc. 1.-3). The three lists do not overlap. In addition, we drew up a list of exons from mono-exonic genes, from protein-coding, and from non-coding genes. These four lists of exons were formatted to enable the comparison of genomic coordinates and to be used as a GTF file with bedtools [35]. The exons from protein-coding genes were extracted from the GTF file describing the complete annotation, and a file containing the genomic coordinates of introns compatible was derived using bedtools analyses.

### 2.4. Annotation/Labelling Methods

To identify exonic circRNAs, we compared the genomic coordinates defined by each circular junction with the boundaries of described exons. Only exact matches between a circular junction and exon boundaries including strand information were used to identify an exonic circRNA. 

To identify intron-derived and sub-exonic circRNAs, we used bedtools [35], in particular bed intersect processes, with two BED files. To identify sub-exonic circRNA, we retained reads containing a circular junction where both segments were mapped inside the exonic region of mono-exonic genes. To identify intron-derived circRNAs, we began by retaining reads containing a circular junction where both segments were mapped inside an intron and ran a selection of these reads compatible with the characteristics of intronic circRNA or intron circles (for details, see Supplementary Doc. 1).

## 3. Results

### 3.1. Selection of Reads for Analysis

As with any transcript, many reads were generated from circRNA, but only reads spanning the circular junction can be used to characterize circRNA. As our main objective was to explore the diversity of production of circRNAs, we considered that each read containing a circular junction deserved consideration as possibly describing a circularization event. The originality of our approach is not the detection of reads containing a circular junction but rather how to manage possible false positives. Our aim was to identify circRNAs that do not originate from a back splicing event. Thus, before being able to detect possible non-canonical circRNAs in the resulting purged list, we first had to identify all exonic circRNAs.

**Figure 1:**
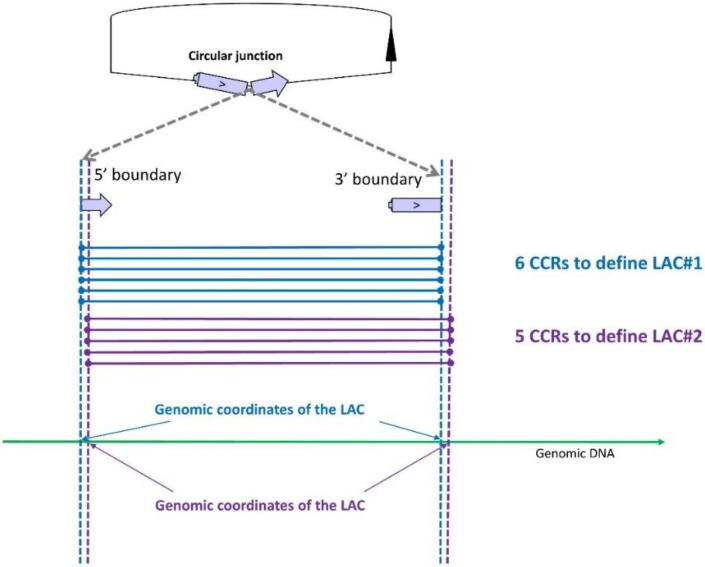
CircRNA production was detected in this region by identifying two clusters of circular chimeric reads (CCRs). The first (in blue) and the second (in purple) contain six and five CCRs, respectively. The two fragments of each CCR are mapped in inverted order on the chromosome. The clustering of CCRs by identical genomic coordinates leads to two loci associated with circRNA production (LAC). Two LACs were thus observed, even if their genomic coordinates differed by only one nucleotide. Contrary to most authors, we do not consider that each LAC defines a distinct circRNA. When we examined the sequence contents of CCRs from different LACs, we observed that several LACs can describe a single circularization event (a single circRNA).

Our approach is based on split alignment as defined by Gao et al. [36]. The approach was originally proposed by Memczak et al. in 2013 [18] but was adapted to currently available alignment tools by Cheng et al. [31]. The reads containing a circular junction can be mapped by STAR [28] and STAR calls them ‘chimeric reads’ (CR) [3,14,37] mapped in two segments. Only when a CR contains a circular junction, hereafter termed ‘circular chimeric reads’ (hereafter CCRs), are the two fragments mapped in inverted order on the chromosome and enable direct identification of the genomic boundaries of the circularized transcript (Figure 1). We assume that clustering CCRs using exactly the same genomic coordinates identifies ‘loci associated with the production of circRNAs’ (hereafter LACs) (Figure 1). In contrast to most authors, we do not consider that this list constitutes a list of distinct circRNAs, but are extremely cautious because we know that reads containing a circular junction originating from an intronic lariat circRNAs are not perfectly mapped by STAR and can lead to several LACs (Figure 1) [14]. When we examined the sequence contents of CCRs at different LACs, we observed that several LACs can describe a single circularization event (a single circRNA) and one LAC can define two or three circRNAs (see below).

Since the objective of this study was to study non-canonical circRNAs and circRNAs produced by non-canonical genes, it was important to choose a high quality dataset. In particular, we wanted to avoid working with artificial reads from the ligation of small RNA fragments. After mapping with STAR, we selected datasets containing a very small proportion of STAR-mapped reads to ‘too many loci’ (<0.03%) and a very small proportion of unmapped reads (<3%). In addition, we chose a dataset that is known to contain a large number of circRNAs including about one hundred of intron-derived circRNAs [14] (SRX5055429). We noted that this dataset [14] features a high proportion of reads mapped by STAR to ‘multiple loci’, as is the case frequently seen in datasets generated after RNaseR [38]. In this dataset, more than 1.5 million CRs identified by STAR and 544,011 were retained as CCRs mapped on porcine autosomes SSC1 to SSC18. Their clustering led to the characterization of 148,505 LACs supported by between one and 7,388 CCRs (average 3.7 CCRs). A large proportion of LACs were defined by only one or two CCRs (Appendix A – Figure A1). 

**Figure A2:**
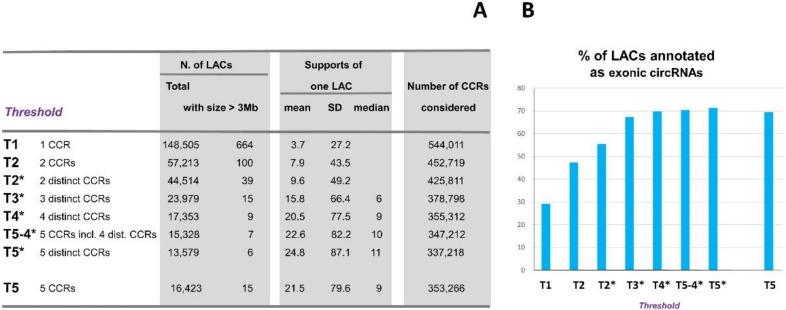
Eight thresholds including the possible consideration of distinct CCRs were tested (**A**,**B**). The seven first thresholds are presented in order of increasing stringency, and the last is the previously used threshold (‘5 CCRs’) [13]. The number of LACs obtained for each threshold is reported. As only 12 genes between 1 and 2 Mb in size have been reported in pig and the length of the longest human gene is 2.3 Mb, we considered that a LAC greater than 3 Mb could be suspicious, and their number is thus reported. (**B**) In each of the eight thresholds explored, we evaluated the proportion of LACs corresponding to the exact back splicing between (one or) two known exons (Ensembl coding genes).

**Figure A3:**
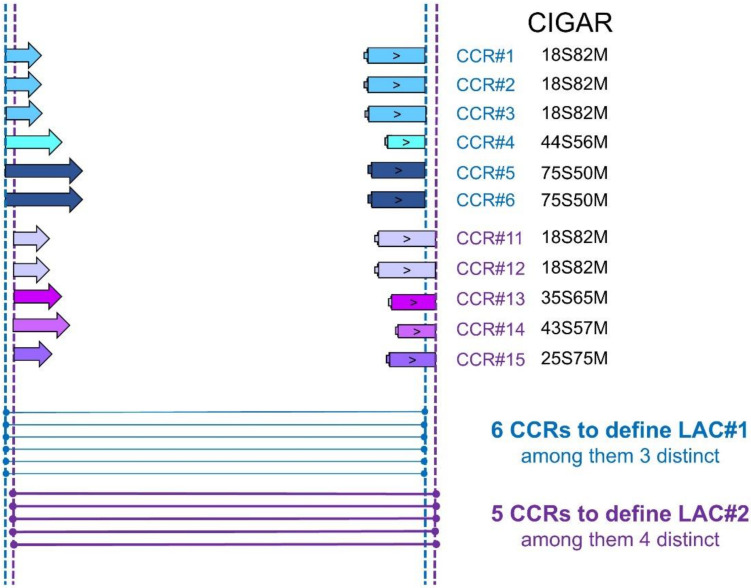
CircRNA production was detected in this region by identifying two clusters of CCRs. The first (in blue) and the second (in purple) contain six and five CCRs, respectively. After clustering of CCRs by perfectly identical genomic coordinates, two loci associated with circRNA production (LAC) were observed, but with a threshold of ‘5 CCRs including 4 distinct CCRs’ only the LAC#2 indicated in purple was retained.

The relevance of LACs supported by only one or two CCRs is questionable and could lead to to many false positive circRNAs. Consequently, in our approach, we introduced an arbitrary threshold for the analysis of the list of clusters of CCRs, which, we believe, is the only way to avoid including false positives. Before defining our threshold, we carefully tested eight possible thresholds (Appendix A, Figure A2) including the possible consideration of distinct CCRs (Appendix A, Figure A3). This criterion was originally suggested to establish a low threshold to avoid considering circRNAs supported by only duplicate reads [18,39]. Finally, we chose only LACs characterized by at least ‘5 CCRs including 4 distinct CCRs’. We retained 15,328 LACs supported by 347,212 CCRs. These LACs were identified by an average of 20 CCRs each and the majority by more than 10 CCRs (see details in Appendix A).

### 3.2. Towards Exhaustive Characterization of CCRs

Based on this selection of LACs characterized by at least ‘5 CCRs including 4 distinct CCRs’, we used a cascade method to investigate the genomic origin of each LAC. The aim was to purge our list of LACs originating from circRNAs that were already described before exploring the potential existence of new classes of circRNAs. We started by exhaustive labelling of LACs originating from back splicing between (one or) two exons, continued by identifying the two types of intron-derived circRNAs, and ended by investigating a possible new class of circRNAs.

#### 3.2.1. Iterative Strategy to Labelling all Exonic circRNAs

We define an exon as a transcribed region embedded in a linear transcript. As our objective was exhaustive labelling of exonic circRNAs, we began by working with a most exhaustive possible lists of exons. We used the complete porcine annotation proposed by Ensembl, plus the list of exons from lncRNA transcripts completed by those in the ALDB database [32,33]. We also drew up a list of possible novel exons from our own datasets (total-RNA-seq and mRNA-seq) from three animals. Although this was only a preliminary step aimed at discarding LACs originating from back splicing, we wanted to specifically evaluate the production of circRNAs from lncRNA. 

To identify all LACs originating from back splicing between (one or) two exons, we used an innovative iterative strategy that goes beyond the original lists of exons (Supplementary Doc. 2A). We started by identifying LACs whose genomic coordinates exactly matched one or two boundaries of previously described exons. This step corresponds to the standard annotation method. After the first round, some circRNAs have one known boundary and one undescribed boundary we used as the new borders in the second round of identification. We hypothesized that these are the frontiers of uncharacterized exons. We repeated this round of identification as many times as necessary.

We performed this original iterative strategy (Supplementary Doc. 2A) using the three lists of exons successively to purge our list of LACs of all circRNAs from the first list before starting to identify on the second list. With this iterative approach, 14,514 LACs were labelled as exonic circRNAs by using the three lists of exons, whereas only 13,142 of them would have been identified as exonic circRNAs if only the standard annotation method using the same three lists had been applied. We noted that in the vast majority of exonic circRNAs, each circRNA is supported by a single LAC.

#### 3.2.2. Intron-Derived circRNAs

**Figure 2:**
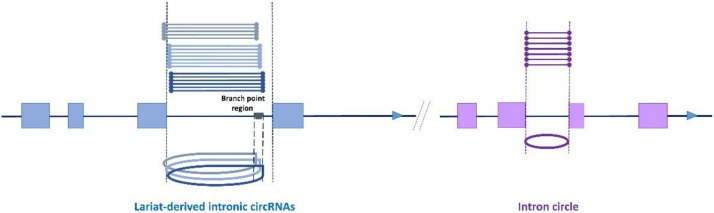
Intron-derived circRNAs. There are two intron-containing genes on this pseudo chromosome. The two multi-exonic genes contain four exons (blue boxes and purple boxes respectively). The analysis of reads (drawn above the chromosome) mapped in this region identified several reads containing a circular junction (CCR). Clustering CCRs allowed us to define several LACs suspected of being associated with different circRNAs. Concerning the gene in blue, three LACs were identified, and their genomic coordinates appeared to be compatible with the production of three intronic circRNAs. Nevertheless, regarding the number of introns able to produce these circRNA, the number was only one. Concerning the gene in purple, one LAC was identified, and its genomic coordinates appear to be compatible with the production of an intron circle. To emphasize the fact that in intronic circRNAs, the circular junction is a 2′-5′ bond, we avoided representing them as a simple circle like intron circles.

We started detecting intronic circRNAs after the list of LACs was purged of all possible exonic circRNAs originating from back splicing in multi-exonic genes. Even though many studies provide lists of ‘intronic circRNAs’ consisting of circRNAs that mapped in intronic regions without the need for further investigation, we consider that the features of circRNAs deriving from intronic lariats were described by Zhang et al. [13]: the first boundary of the circular junction must coincide with the beginning of the intron and the second boundary must be compatible with a circularization event limited by the branch point (Figure 2). Only coding genes from Ensembl were used for the identification of intron-derived circRNAs. 

**Figure 3:**
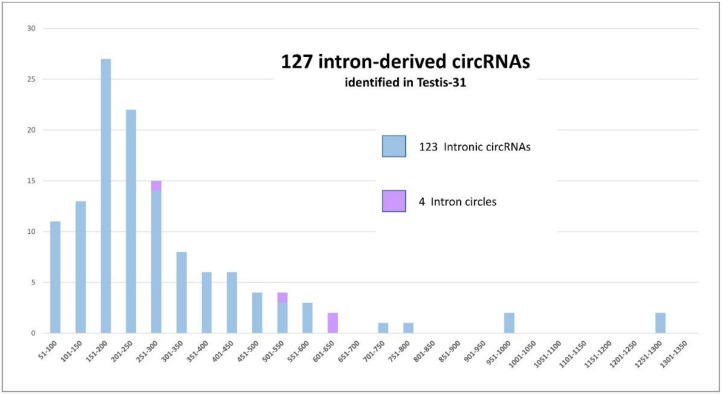
The height of the bars represents the size of the 127 intron-derived circRNAs identified in the Testis-31 dataset. A total of 123 lariat-derived intronic circRNA (in blue) and 4 intron circles (in purple) were detected in the dataset. The circRNAs were divided into groups according to their size (*x* axis) and the number of circRNAs concerned is shown on the *y* axis.

We began the analysis by searching for lariat-derived intronic circRNAs using the genomic coordinates of the LACs. We identified only 93 of the previously detected 118 intronic circRNAs [14]. Thirty new intronic circRNAs were identified. These results can be explained by the ‘5 CCRs including 4 distinct CCRs’ threshold, which was stricter than the one used previously and was applied earlier in the process of selecting CCRs [14]. Moreover, this characterization was performed without the previously applied restrictions on circRNA size [14]. The second type of intron-derived circRNA, intron circles, contain the entire intron sequence (Figure 2) [28,29]. All LACs originating from intron circles were identified. Three previously described [14] and one new intron circle were detected. For these intron-derived circRNAs, the number of introns able to produce intron-derived circRNA was less than the number of LACs (see Figure 2). The sizes of the 127 intron-derived circRNAs are analyzed in Figure 3. The smallest was an intronic circRNA of 78 nucleotides that originated from *THBS3*. The average size of intronic circRNA was 280 nt and less than 5% contained more than 600 nt.

**Figure 4:**
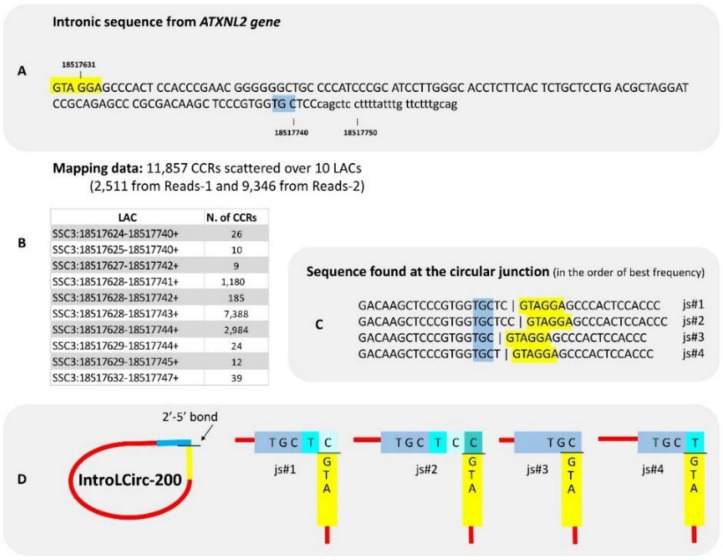
introLCirc-200. (**A**) The intronic sequence concerned by production of this circRNA. This intron is framed by two exons of 213 bp (ENSSSCE00000205149) and 190 bp (ENSSSCE00000199966 or the 3′ UTR exon). (**B**) 10 LACs were characterized. When we examined the sequence of CCRs and their mate pairs, four distinct ‘junction-sequences’ (js#1-4) were identified at the circular junction (**C**,**D**). They are presented in decreasing order of frequency. For details, see Supplementary Doc. 3A1.

Among lariat-derived intronic circRNA, IntroLCirc-200 was identified from the intron of *ATXN2L* gene (Figure 4A) with a very large number of chimeric reads: 11,857 CCRs distributed over 10 LACs (114–117 nt), the boundary positions differed of which by only a few bases (Figure 4B). If we consider that the 10 LACs are located on the same locus, it is the locus that produces the largest number of circRNA copies in this data set. Its size appears small compared to the length of reads (2 × 100 and 2 × 125 bp) and we expected to find CCRs in both Reads-1 and Reads-2. We were surprised to observe 2511 in Reads-1 and 9346 CCRs in Reads-2. The deficit in CCRs in Reads-1 can be explained by unreliable sequencing in a GC-rich part of IntroLCirc-200 (Supplementary Doc. 3A1). When we considered the sequence of all CCRs and their mate pairs, the circular structure was confirmed and four distinct ’junction-sequences’ were characterized at the circular junction (Figure 4C,D). We also observed minor differences near the circular junction between the Reads-1 and the Reads-2 of a pair. These differences were due to the limited fidelity of the reverse transcriptase near a 2′-5′ link [9].

#### 3.2.3. A New Class of Exon-Derived circRNAs: Sub-Exonic circRNAs

At this point, the list of LACs was purged of all previously described classes of circRNAs. When we examined the list of unallocated LACs, which was very short (<700 LACs), we noticed a large number of LACs (45) spanning the single exon of *RNase_MRP*. Consequently, we decided to identify circRNAs produced by mono-exonic genes and that included only part of the exon. The possible production of further circRNAs was observed in 19 genes. The dataset contains a mix of 2 × 100 and 2 × 125 bp and the mapping by STAR retained CCR only if they included two segments. Consequently, we decided not to retain LACs of less than 55 pb and only 18 genes remain suspected of producing sub-exonic circRNAs including two coding genes (Supplementary Doc. 3B).

**Figure 5:**
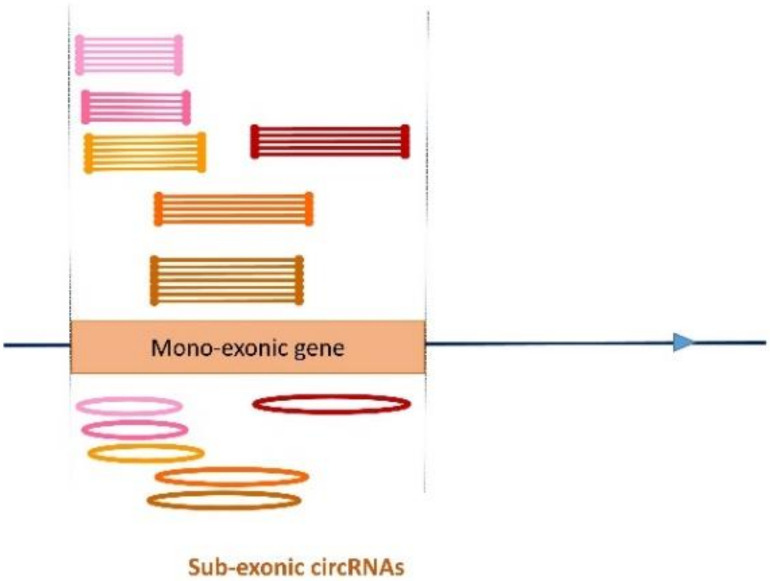
Sub-exonic circRNA. Clustering CCRs allowed us to define several LACs suspected of being associated with different circRNAs. On this pseudo chromosome, we show a mono-exonic gene able to produce several sets of sub-exonic circRNAs. Six LACs were identified, whose genomic coordinates appear to be compatible with the production of several sub-exonic circRNAs. When we examined the sequence contents of CCRs from different LACS, we saw that several LACs described a single circularization event (a single circRNA) or that one LAC can define two or three circRNAs.

We propose to call this new class of circRNA that includes only part of the exon ‘sub-exonic circRNAs’. In 11 out of 18 cases, several different sized LACs were observed for a single exon (Figure 5).

**Figure 6:**
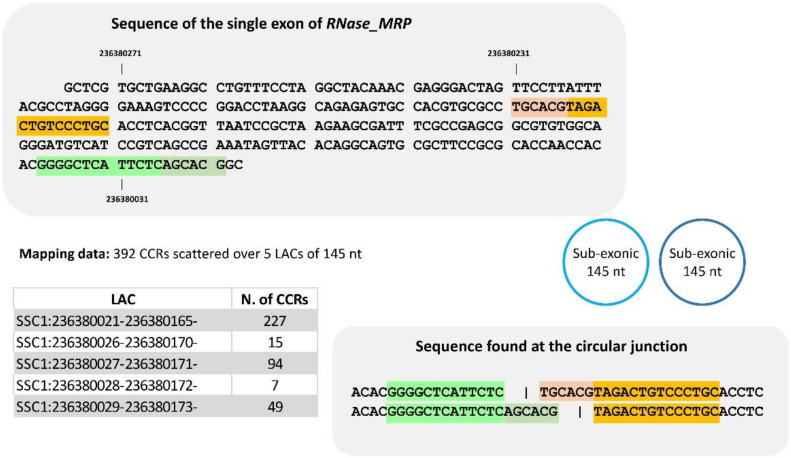
Analyses of sequence at the circular junction of a set of sub-exonic circRNA produced by *RNase_MRP*. The set is defined by five LACs of 145 nt. When we examined the sequence of CCRs and their mate pairs, two distinct ‘junction-sequences’ were identified at the circular junction, respectively. This is an example of several LACs defining a single circRNA. For details, see Supplementary Doc. 3A2.

To improve our knowledge of sub-exonic circRNAs and because some are very small, we examined the CCRs and their mate pairs identified in three sets of LACs from the *RNase-MRP* (nc gene, SSC1). Our examination of the sequence content of the reads of the first one comprising five LACs (145 nt), leads us to propose only two distinct sub-exonic circRNAs (145 nt) (Figure 6, and details in Supplementary Doc. 3A2). For the second one identified by three LACs (115 nt), all reads examined were compatible with a single sub-exonic circRNA including 115 nt (Supplementary Doc. 3A3). In the last region examined (Supplementary Doc. 3A4), sub-exonic(s) circRNA(s) of 61 nt was suspected. We were surprised to see that a large proportion of CCRs were 125 bp long (the dataset contains a mix of 2 × 100 and 2 × 125 bp). Consequently, one would expect to find the circular junction of such a small circRNA (61 nt) twice in a read of 125 bp, which would give rise to more than two segments. Among the CCRs with a length of 125 bp, we found sequences that are not compatible with the hypothesis of a circRNA of 61 nt (Supplementary Doc. 3A4). In these sub-exonic circRNAs, we observed shifts in alignment in identical sequences and the number of distinct circRNAs was less than the number of LACs.

### 3.3. Comprehensive Overview of circRNAs from Non-Coding Sequences

#### 3.3.1. Exonic circRNAs from Non-Coding Genes?

Our methodology included exhaustive labelling of exonic circRNAs based on an iterative strategy whereas only 90% of circRNAs would have been identified using the standard annotation methodology (13,142/14,514). Rather than analyzing each specific exonic circRNA included in these lists to search for its parent gene, we performed a generalized analysis of the population of circRNAs present in these lists and of their origin.

The 14,514 exonic circular RNAs originating from multi-exonic genes were supported by 319,453 CCRs (92.0%) retained for Testis-31. A total of 281,790 CCRs (81.16%) can be assigned to the production of exonic circRNA from the multi-exonic protein-coding genes, 19,646 (5.66%) from lnc and 18,017 (5.19%) from other multi-exonic genes (Supplementary Doc. 2B1). These results were obtained with the Testis-31 dataset [14]. We then combined CCRs from datasets obtained from Testis -05 (SRX5055428) and -54 (SRX5055430) to simulate a second independent dataset with 11,343 LACs defined by 210,875 CCRs. The analysis of this combined dataset produced very similar results (Supplementary Doc. 2B2). We suggest that from 5.7% to 11% of the CCRs could thus have originated from lnc RNA genes in Testis-31.

We then analyzed the 14,514 exonic circRNA we identified to see if we could find any exonic circRNAs originating from pseudogenes (126 are described in pig [27]) but obtained no results. We performed an analysis to identify exonic circRNAs originating from mono-exonic nc genes (2800 described in pig [27]). Three LACs corresponding to three exonic circRNAs produced by three snoRNAs were identified (Supplementary Doc. 3B). In addition, two LACs that were identified in this way originated from *SNORD104*. Curiously, 70 CCRs (all from Reads-1) defined the first LAC and 84 CCRs (all from Reads-2) defined the second LAC, both 71 nt in size. Nevertheless, we found only 86 distinct PE reads, plus all reads contained the back junction (see details in Supplementary Doc. 3A5). When the sequence content of these reads was examined, it appeared to be compatible with an exonic circRNA including 71 nt (reported in Supplementary Doc. 3B). The detection of two LACs was due to a shift in alignment: two bases present at the circular junction may be aligned on both exonic borders and STAR did not produce the same read map when the CCRs came from Reads-1 rather than Reads-2 and vice versa. We observed that all the CCRs were 100 bp long whereas 3/4 of the reads in this dataset were 125 bp long. As explained above, this observation is not surprising, and is one more reason to believe that the characterization of this very small circRNA is reliable.

Among the 2800 mono-exonic RNA genes described in pig [27], we only characterized four as being able to produce exonic circRNA (Supplementary Doc. 3B). Concerning exonic circRNAs originating from lncRNA, we suggest that only 5.7% to 11% of the CCRs considered could originate from lncRNA genes in Testis-31.

#### 3.3.2. Non-Canonical circRNAs

When we analyzed the splicing signal detected by STAR in the borders of the circular junction of the 10,797 exonic circRNAs shown to result from back splicing between (one or) two exons reported by Ensembl (coding genes), we found that only 0.13% of the circular junctions were non-canonical splice junctions. When STAR analyzed the sequence at the edge of the circular junction of LACs characterizing intronic and sub-exonic circRNAs, it also almost never detected the canonical splicing motifs (GT/AG) (99% and 97%), evidence that using this filter would be counterproductive for the detection of circRNAs other than exonic ones. 

We examined the list of unallocated LACs, which was now very short, and six further LACs associated with a large number of CCRs caught our attention as deserving investigation (highlighted in yellow in Supplementary Doc. 3B). In five of them, these new transcripts appeared to be non-coding and mono-exonic. Closer examination of their sequences showed that two could have derived from ribosomal RNA genes. In three, we observed the production of sub-exonic circRNAs and in the last two, we suspected the production of exonic circRNAs. The sixth appeared to be located inside an intron of a gene (*PKN3*) that may consequently be incorrectly annotated, and Split Reads (SR) reported by STAR in this region support this hypothesis. This LAC appears to be associated with a lariat-derived intronic circRNA.

The most abundant circRNA, IntroLCirc-200, in porcine pubertal testis (in Testis-31 and in the combined dataset) was a lariat-derived intronic circRNA from *ATXN2L*. If we had not encountered a problem of unreliable sequencing in the GC-rich part (Supplementary Doc. 3A1), we might have found even more CCRs at this locus. In humans, the testis is the tissue with the highest level of expression of this gene [40]. In porcine testis-31, the expression level measured in the mRNA-seq (RSEM) is ranked 394th out of 18,057 genes expressed. These data are compatible. Next, we compared the number of linear transcripts with those of circular intronic transcripts produced by this gene in the Testis-31. We calculated the quantity of *ATXN2L* linear transcripts through the number of SRs (287) observed at this exon exon junction and the quantity of intron-derived circRNA through the number of CCRs observed for IntroLCirc-200 (11,857) in the total-RNA-seq. This comparison clearly showed that this circular transcript is much more abundant than *ATXN2L* linear transcripts. This is the first time we have found such a marked imbalance (CCR/SR=41) in favour of an intron-derived transcript in this dataset (Testis-31) [14].

The production of sub-exonic circRNA was observed in only 21 mono-exonic genes (Supplementary Doc. 3B). Of the 21, 16 have already been described as short non-coding genes and three appeared to be new nc genes (including two probable ribosomal RNAs). Two have previously been described as protein coding genes but one of the two was deleted from the new release of Ensembl [27] and the second was identified by only five CCRs organized in a single set. Among the 21 genes identified as being able to produce sub-exonic circRNA, the most convincing are listed in Table 1, and we suggest retaining only LACs up to 70 nt in size.

In sub-exonic and intronic circRNAs, we observed several circularization events that differed only in a small number of nucleotides, and in addition, we frequently observed alignment shifts, thus there were fewer distinct circRNAs than LACs. This underlines the fact that most non-canonical circRNAs originate from non-coding sequences, either introns or non-coding genes. Concerning the non-canonical circRNAs, several LACs described a single circularization event (a single circRNA) and one LAC described two or three circRNAs.

## 4. Discussion

Our main objective was to explore the diversity of circRNA production rather than to provide a comprehensive list of circRNAs or a new pipeline for their detection. We think that each read containing a circular junction deserves to be considered as describing a circularization event as long as this event occurred several times. We chose to not perform a selection likely to impact certain classes of circRNAs such as filtering the canonical splicing motifs. Our study has shown that using this filter on the canonical splicing motifs can have a very negative influence on the detection of non-canonical circRNAs. Although many authors consider this filter a good way to eliminate false positives, in fact they are only false positives with respect to the exonic circRNA model. A number of bioinformatic approaches have been proposed to detect circRNAs that focus mainly on exonic circRNAs [36,41]. To detect non-canonical circRNAs, we suggest using a minimum selection strategy based on a search for all possible back fusion points that are captured in RNA-seq data.

To avoid including false positives in our analysis, we disregarded very rare circularization events. In the literature, definitions of a locus suspected to produce a circRNA vary but none are supported by extensive evaluation. The most frequent choice is ’two CCRs’ [22,42] or ’two distinct CCRs’ [18,39] but some authors underline the need for a stringent choice [14,43] while others appear to considerer all CCRs with no threshold [24,25]. The real novelty of the current study is therefore the approach chosen to determine this threshold. We believe that possible false-positives are not among those resulting from the same circularization event that occurred several times and for which we can propose a model for their biogenesis. We are aware that the limit between sporadic circularization events and the real production of circular transcripts is very difficult to capture, but we believe that this choice enabled us to discard the great majority of false positive identifications of circRNA. We are confident that this strategy (i.e., a high threshold but no additional filter) has no negative impact on the characterization of exonic circRNAs, quite the contrary, it makes it possible to identify other circRNAs.

In contrast to the usual alignment of read pairs from paired-end data [41], we aligned each mate read separately. In contrast to the DCC pipeline [31] and to the method we used in our first study [14], we completed our characterization of circRNAs without using mapping information concerning the second mate in the pair. The example of IntroLCirc-200 (Figure 4, Supplementary Doc. 3A1) shows the merit of not systematically rejecting the CCR when mapping the second mate does not agree with the CCR map. The polymerase used in sequencing is less reliable in GC-rich regions and such a filter could thus affect the characterization of non-canonical circRNAs. As underlined by Gao et al. (2018) [36], the aligner can facilitate circRNA detection by providing accurate and comprehensive mapping information. In our analysis, the aligner does most of the work involved in circRNA detection. We chose the ‘easy way’, i.e., using a splice-aware aligner that was developed specifically for RNA-seq reads across intron-sized gaps on genome references. Now that we know that these non-canonical circRNAs are derived from non-coding sequences, doing the initial mapping with STAR may be called into question. 

In the present study, all reads containing a circular junction were considered without applying a minimum size for putative circRNAs. We do not know the size, but except for non-canonical circRNAs, we can assume it coincides with the genomic size of LACs. Previous authors characterized intron-derived circRNAs with a minimum size of 140 [14] or 200 nt [13]. The data presented in this paper show that the characterization of very small circRNAs (<100 nt) is possible but should be undertaken with extreme caution (and no blind confidence in the mapping of the respective CCRs by STAR). The impacts of introducing a minimum genomic size would be very limited when the study focuses on exonic circRNAs but the present study underlines the importance of this parameter for non-canonical circRNAs. 

With the current work, we do not claim to have studied circRNAs originating from lncRNA, and our knowledge of them at this point is still too preliminary (only 316 described in pig [27]) to undertake complete identification of exonic circRNA from lncRNA. We simply suggest that 5.7% to 11% of the CCRs considered could originate from lncRNAs in Testis-31 and consequently, that these genes should be considered as capable of producing exonic circRNAs like other pluri-exonic genes. The ability to produce at least two exonic circRNAs does not differ between coding (45%, previous study [14]) and long non-coding genes (50%, data not shown). Moreover, the absence of a correlation between the production of poly-adenylated linear transcripts and exonic circRNA is a feature shared by coding [14] and non-coding (Supplementary Doc. 2C) genes. We believe it is important to emphasize that transcripts from lncRNA genes present some particularities. We have very frequently observed that two or three different borders are proposed in databases for the beginning or the end of an exon involved in lncRNA. Given the poor current state of our knowledge about the annotation of lncRNAs, we preferred to not investigate circRNA derived from lncRNA introns.

On the other hand, we identified genes able to produce circular RNAs from an exon that does not correspond to the exact circularization of the exon. A sub-exonic circRNA contains only part of the exon and, in most cases, we observed several circRNAs originating from an exon (organized in sets). This study showed that 16 known RNA genes are able to produce sub-exonic circRNAs and we identified three new loci based on their ability to produce sub-exonic circRNAs. These 16 genes have to be contrasted to the 2800 mono-exonic and non-coding genes described in pig [27]. We showed that mono-exonic and nc genes are able to produce sub-exonic circRNAs and consequently that a feature of sub-exonic circRNAs could be their small size (<400 nt) [21]. The GC content of the exon sequence does not seem to be decisive for the production of sub-exonic circRNAs (Table 1). The transcription of these genes does not require the splicing step and we suggest that sub-exonic circRNAs are the first circRNA produced independently of the splicing of the pre-messenger. Since it is difficult to talk about splicing for a transcript of a mono-exonic gene, it is also difficult to talk about back splicing or to suggest that these sub-exonic circRNAs define new exons. Apropos, we can ask ourselves which elements should be used to define an exon, particularly when the gene is mono-exonic. This study has shown that identifying circRNA is a very promising way to advance our knowledge of these non-coding genes.

We characterized sub-exonic circRNA produced by nc genes in porcine species but the interest of this study goes beyond the pig genome. We note that the production of circRNA by the mono-exonic gene *RNase_MRP* was also reported in humans by Liu et al. [44]. Using a similar method to the one we used in the present study, these authors identified a group of *interior* circRNAs. As we did, they identified reads containing circular junctions using the strategy proposed by Memczak et al. [18] neither did they add any filters. Liu et al. proposed that the group of interior circRNAs is formed according to their overlap with known genetic components such as exons, introns, and intergenic regions. We believe that our approach is more rigorous because we did not include sporadic circularization events, we labelled as many exonic circRNAs as possible, and also identified intronic circRNAs and intron circles before examining the composition of the group of unallocated circRNAs.

The list of mono-exonic genes able to produce sets of sub-exonic circRNAs is very short (two ribozymes, nine snoRNA, one snRNA, one scaRNA, three misc_RNA and three new genes including two ribosomal RNA genes). The production of circular transcripts by ribosomal RNAs has never been properly explored in eukaryotes and this study simply shows that it may not be equal to zero. As is usually the case in circRNA studies, the datasets are derived from the sequencing of total RNAs after depletion of ribosomal sequences. Even more rarely, ncRNA can produce mono-exonic circRNAs (four snoRNAs and two new genes). In a study exclusively dedicated to exonic circRNA, Kaur et al. (2018) [22] already reported exonic circRNAs produced (66–135 nt) by four snoRNA genes in humans. Nevertheless, we suggest it would be wise to avoid rapidly coming to the conclusion that the production of circRNA by mono-exonic genes is marginal: knowledge concerning short non-coding RNA (which are mono-exonic genes) is probably biased by current sequencing techniques and gene annotation [45]. 

We characterized a total of 123 introns as being able to produce circRNA. This number contrasts the more than 450,000 introns produced by protein-coding genes described in pig [27]. The 123 introns we identified are capable of producing an intronic circRNA, i.e., a circular transcript derived from the intronic lariat. We know that the intronic lariat is only an intermediate molecule that is usually rapidly degraded, and that degradation is initiated by cleavage of their internal 2′-5′ phosphodiester bonds by a unique debranching endonuclease [46]. Some introns appear to avoid this turnover pathway and form stable intronic sequence RNA (sisRNA) [47,48]. This escape may be linked to the absence of an adenosine at the branch point, a feature frequently observed in introns able to produce circRNAs [12,13,14] and also observed in the present study with IntroLCirc-200 (Figure 6). In porcine pubertal testis (Testis-31, -05, -54), the circRNAs associated with the highest number of reads is the lariat-derived intronic circRNA from *ATXN2L* (IntroLCirc-200). We are convinced that this presence is not only due to the exceptional stability acquired by circularization. In 2013, Hesselberth suggested [46] that many introns have been evolutionarily repurposed to serve roles after splicing. By studying sisRNA in *Drosophila melanogaster*, Jia Ng et al. [49] suggest that some introns could be transcribed independently of the gene. Even if this suggestion referred to polyadenylated sisRNA, it could equally apply to some circular sisRNA such as IntroLCirc-200. To explore this hypothesis, we investigated the balance between the intronic circRNAs and the linear transcript of *ATXN2L* (accessible by the ratio CCR/SR). This ratio was estimated to be 41 for Testis-31 but only 2.4 and 3.9 for Testis-54 and Testis-05 respectively. Moreover, the number of SRs observed for the corresponding exon-exon junction was lower than the others for this gene (436 on average for four upstream exon-exon junctions and 287 for this one, Testis-31). The analyses of these ratios suggest that the production of IntroLCirc-200 is regulated. We suggest that the intron producing IntroLCirc-200 is an example of sisRNA, which is transcribed independently of the *ATXN2L* gene. We suggest considering this sisRNA as the product of a new non-coding RNA gene. The current state of knowledge suggests that only RNA genes located outside an intron can be independently transcribed by the RNA polymerase III [21]. The production of such embedded nc RNA not only requires parental gene transcription by the RNA polymerase II, but also splicing of the corresponding intron and removal of the excess intronic sequences. Usually these intronic nc RNAs are not capped or polyadenylated in their mature form. Current knowledge concerning this possible class of gene is therefore a little contradictory depending on whether we look at the sisRNA side or the ncRNA expert side. Here, it is very probable that the main transcript of this gene is circular, which could have two advantages: (1) being circular enables resistance to exonucleases, (2) the joining of the ends could create a particular sequence and the 2′-5′ link a particular structure that could serve as a recognition motif. The hypothetical second advantage could be challenged since we have not been able to rule out the possibility there are several independent circularization events (Figure 4). We are convinced that this intronic transcript originating from *ATXN2L* has a particular function in porcine pubertal testis, at least at one point in time. We may be witnessing the emergence of a new non-coding gene in the porcine genome.

This study was performed using a porcine dataset and the observations reported here on intronic circRNA from *ATXN2L* are very probably a feature of pigs. Nevertheless, our study shows the interest of investigating intron-derived circRNA. Indeed, the 2′-5′ links, probably associated with a strong intronic RNA structure, complicate their detection using standard techniques, and their abundance is consequently often underestimated. Intronic lariat-derived circRNAs are the oldest non-canonical circRNAs described, and even though their large-scale study will require overcoming some biological barriers, we hope that this study will inspire others to investigate this major class of non-canonical circRNAs.

As the objective of this study was to investigate non-canonical circRNAs and circRNA produced by non-canonical genes, we chose to work on datasets generated by sequencing stranded RNA from healthy testes. These represent rigorous choices, as was the choice to proceed by annotation rather than by simple classification based on mapping information as first proposed by Memczak et al. [18]. The only disadvantage of working with data from pig is the lack of comprehensive lncRNA annotation in the Ensembl database for this species, but this can be compensated by using others databases. We found a real advantage in working with the porcine genome, because there is a unique status assigned to each gene in the porcine genome, whether its transcripts are classified as ‘lnc’ or ‘coding’s. In pigs, there are no anti-sense (lnc) transcripts assigned to a coding gene. It is probably not the true situation, but it enabled us to avoid describing a lot of irrelevant anti-sense circRNAs. 

Our main objective here was to characterize circRNAs that do not originate from back splicing between exons. Even though we are aware that the tools available for the detection of circRNAs may not appear to be suitable for the identification of the non-canonical circRNAs, and even though the strategy implemented here (STAR, high threshold but no additional filter) seems to have no negative impact on the characterization of exonic circRNAs, there is still a long way to go before we can propose a bioinformatic approach that is appropriate for all circRNAs. The quality of the sequences involved in the non-canonical circRNAs (non-coding sequences) and their size are major obstacles to their identification and for the relative quantification of their presence. Now that we know these non-canonical circRNAs are derived from non-coding sequences, the choice of the best initial aligner may seem questionable. It is also important to underline the biological obstacles associated with the 2′-5′ bond present in intronic circRNAs [14]. In this study, we chose to first exclude exonic circRNAs by comprehensive CCR assignment to exons in order to focus on these non-canonical circRNAs. We are now in a position to suggest an alternative methodology, which would consist of removing exonic circRNAs using the filter based on the canonical splicing patterns and to make a selection based on the size of the LACs (<800 bp).

The ‘wide-angle’ approach, to this study gave us the opportunity to make non-canonical observations about circRNAs, thereby advancing our knowledge of intronic circRNAs, in addition to which we identified a new class of circRNAs we call sub-exonic circRNA. Our study emphasizes the likelihood that analyzing all circRNAs, and particularly those least studied so far, will significantly improve our knowledge of non-coding genes. 

## Figures and Tables

**Table 1:** Selection of the examples that most convincingly support the hypothesis that mono-exonic genes (non-coding) able to produce sub-exonic circRNAs. The statistics reported concern testis-31 but all are present in the combined dataset. If Only LACs > 70 nt were considered but only sub-exonic circRNAs from RNase_MRP were impacted. * see details in Supplementary Doc. 3B–C

Set of LACs	N. CCR	Gene_ID	Gene Biotype		Exon	GC %
15 × 107–124 nt	503	ENSSSCG00000018563	snoRNA	*SNORA48*	135 bp	54.0%
8 × 93–138 nt	165	ENSSSCG00000019944	snoRNA	*SNORD97*	141 bp	39.0%
9 × 71–193 nt	79	ENSSSCG00000040361	spliceosomal RNA	*U3 *	216 bp	49.0%
38 × 91–199 nt	1750	ENSSSCG00000018700	ribozyme	*RNase_MRP*	258 bp	58.5%
9 × 134–271 nt	294	ENSSSCG00000020439	ribozyme	*RNaseP_nuc*	327 bp	61.7%
7 × 76–144 nt	83	ENSSSCG00000040520	snoRNA	*SCARNA10*	328 bp	48.3%
5 × 350–358 nt	122	novel nc-SSC1:65kb			360 bp	61.7%*
20 × 88–244 nt	1016	novel nc-SSC7:10Mb	prob. ribosomal		515 bp	56.9%*
5 × 102–118 nt	53	novel nc-SSC16:37Mb	prob. ribosomal		125 bp	44.0% *

**Figure A1:**
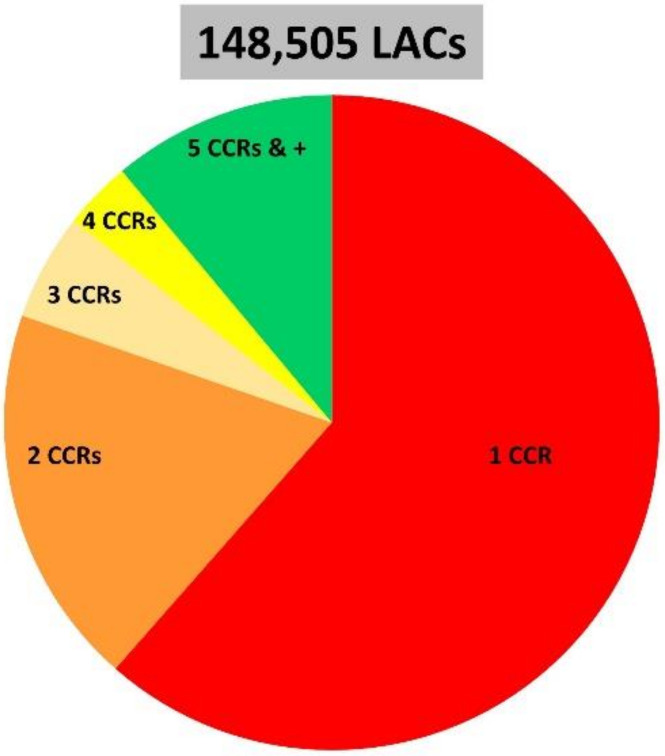
The clustering of 544,011 CCRs mapped on porcine autosomes SSC1 to SSC18 led to the characterization of 148,505 LACs. The pie chart shows the proportion of LAC supported by 1, 2, 3, 4 or 5 and more CCRs.

## Supplementary Materials

The following are available online at https://www.mdpi.com/2073-4409/9/8/1806/s1, Supplementary documents 1 (details of materials and methods); Supplementary documents 2 (details of results concerning exonic circRNAs); Supplementary documents 3 (details of results concerning non-canonical circRNAs)

## Appendix A

In each of the eight cases explored, we evaluated the proportion of LACs corresponding to exact back splicing between (one or) two known exons (Ensembl coding genes [27]). We observed that the proportion of exonic circRNA identified increases with the stringency of the threshold, but that leveling off occurred at around 70%. We think that this stabilization is a good indicator to eliminate the maximum possible number of sporadic circularization events. Among clusters with more than four distinct CCRs, we identified more than 70% as resulting from exact back splicing between exons of genes coding for proteins, whereas for CCRs remaining alone after clustering, the score was only 17.8%.

Among the original 148,505 LACs, we identified 664 LACs that were more than three Mb in size. As only 12 genes between 1 and 2 Mb in size have been reported in pig and the length of the longest human gene is 2.3 Mb, we consider that a LAC greater than 3Mb can be suspected of being false positives. Another indicator of the elimination of a maximum number of false positive could be the decrease of the number of LAC greater than 3 Mb. Among the seven LACs greater than 3 Mb finally retained in this study (T5-4*), we noted that six are on the list of 478 LACs not assigned in this study.

In addition, we suggest considering the mean and the median of the number of CCRs defining LAC.

To avoid considering circRNAs supported by only duplicate reads, we evaluated the number of distinct reads using CIGAR (here we consider that CCR#1/CCR#2/CCR#3, CCR#5/CCR#6 and CCR1#11/CCR#12 contain the same information). This count of distinct reads is capped by the size of the reads and is only of interest for LACs supported by a very small number of CCRs. After applying the threshold to select LACs, only the number of CCR was considered.
