# Supplementary material for: In-Depth Analysis Reveals Production of Circular RNAs from Non-Coding Sequences
Source: Cells. 2020 Jul 30;9(8):1806. doi: 10.3390/cells9081806 (PMC7464727; doi:10.3390/cells9081806)
Supplement: Supplementary file 1 [file cells-09-01806-s001.pdf]

Supplementary Document 1

In this document, we will give a maximum of information about

(1) the sequencing and mapping of reads
(2) the selective process of chimeric reads (CR) mapped in inverted order (or CCRs) and output files
(3) Annotation of exonic circRNAs
(4) Annotation of intron derived circRNAs

1. Sequencing and mapping of Total RNA

For Illumina total RNA sequencing, rRNA depletion was done with the RiboMinus eukaryote kit (ThermoFisher scientific) according to the manufacturer's recommendations. Measurements made with an Agilent Bioanalyser confirmed successful rRNA depletion. The Illumina stranded Total RNA TruSeq library preparation kit was used to generate libraries for stranded paired-end sequencing. After sequencing on HiSeq2500 or HiSeq2500-1T, two types of reads were available (100 and 125 bp from the same library) for each animal [1]. Data were processed to remove adapter sequences and reads with low sequence information.

Reads-2 contain the sequence written in the sense-gene.

Mates of each pair were mapped independently with STAR (STAR-SE), and we decided to search interesting reads from CR alignments. Parameters proposed by Cheng et al. (2016) [2] were used in particular those concerning the chimeric reads ("chimSegmentMin 15", "chimScoreMin 15", "chimScoreSeparation 10" and "chimJunctionOverhangMin 15"). Essentially, we retained chimeric reads with only two segments and with a minimal size for the smallest mapped segment of 15 bp.

All useful information concerning CR was included in annex files (Chimeric.out.sam and Chimeric.out.junction) generated by STAR SE1 and STAR-SE2 mapping. The file SJ.out.tab generated by STAR contains the number of split reads spanning each exon-exon junction. These count tables are simple tab delimited text files that are considerably smaller than traditional aligned BAM files and can be easily processed on desktop computers.

2. Building of the list of CR mapped in inverted order

The two files generated by STAR SE chimeric.out.junction generated for Reads1 and for Reads-2 are used to select CR mapped in inverted order.

column	-1	-2	-3	-4	-5	-6	-7	-8	-9	-10	-11	-12	-13	-14	
line-1	18	41620104	+	18	41623937	+	0	0	1	HISEQ:29:C6	41620061	43M82S	41623938	43S82M	*R2*
line-2	18	41620104	+	18	41623937	+	0	0	1	HISEQ:29:C6	41620061	43M82S	41623938	43S82M	*R2*
line-3	18	55382707	+	18	55373242	+	1	2	0	HISEQ:29:C6	55379665	30M2946N66M29S	55373243	96S29M	*R2*
line-4	18	55382707	+	18	55373242	+	1	2	0	HISEQ:29:C6	55382649	58M67S	55373243	58S67M	*R2*
line-5	18	55382707	+	18	55373242	+	1	2	0	HISEQ:29:C6	55382643	64M61S	55373243	64S61M	*R2*
line-6	18	55373242	-	18	55382707	-	2	0	2	D3VDZHS1:1	55373243	60S40M	55382647	60M40S	*R1*
line-7	18	55373242	-	18	55382707	-	2	0	2	D3VDZHS1:1	55373243	31S69M	55382676	31M69S	*R1*
line-8	18	55540797	-	18	55541187	-	0	0	0	D3VDZHS1:1	55540798	66S34M	55541121	66M34S	*R1*
line-9	18	55540797	-	18	55541187	-	0	0	0	HISEQ:29:C6	55540798	91S34M	55541096	91M34S	*R1*

A small part of the two files chimeric.out.junction obtained from Reads -2 or from Reads-1

The first 9 columns give information about the chimeric junction:

Column-1: chromosome of the donor

Column-2: first base of the intron of the donor (1-based)

Column-3: strand of the donor

Column-4: chromosome of the acceptor

Column-5: last base of the intron of the acceptor (1-based)

Column-6: strand of the acceptor

Column-7: splicing junction type: 0= non canonical intronic motif, 1=GT/AG, 2=CT/AC

Column-8: repeat length to the left of the junction

Column-9: repeat length to the right of the junction

Columns 10-14 describe the alignments of the two chimeric segments. They are SAM like. Alignments are given with respect to the (+) strand

Column-10: read name

Column-11: first base of the first segment (on the + strand)

Column-12: CIGAR of the first segment

Column-13: first base of the second segment

Column-14: CIGAR of the second segment

Both files “chimeric.out.junction” were concatenated, and we keep the information concerning the origin of read in a new column-15.

column	-1	-2	-3	-4	-5	-6	-7	-8	-9	-10	-11	-12	-13	-14	-15
line-1	18	41620104	+	18	41623937	+	0	0	1	HISEQ:29:C6	41620061	43M82S	41623938	43S82M	*R2*
line-2	18	41620104	+	18	41623937	+	0	0	1	HISEQ:29:C6	41620061	43M82S	41623938	43S82M	*R2*
line-3	18	55382707	+	18	55373242	+	1	2	0	HISEQ:29:C6	55379665	30M2946N66M29S	55373243	96S29M	*R2*
line-4	18	55382707	+	18	55373242	+	1	2	0	HISEQ:29:C6	55382649	58M67S	55373243	58S67M	*R2*
line-5	18	55382707	+	18	55373242	+	1	2	0	HISEQ:29:C6	55382643	64M61S	55373243	64S61M	*R2*
line-6	18	55373242	-	18	55382707	-	2	0	2	D3VDZHS1:1	55373243	60S40M	55382647	60M40S	*R1*
line-7	18	55373242	-	18	55382707	-	2	0	2	D3VDZHS1:1	55373243	31S69M	55382676	31M69S	*R1*
line-8	18	55540797	-	18	55541187	-	0	0	0	D3VDZHS1:1	55540798	66S34M	55541121	66M34S	*R1*
line-9	18	55540797	-	18	55541187	-	0	0	0	HISEQ:29:C6	55540798	91S34M	55541096	91M34S	*R1*

Now we select lines with c1=c4 and c3=c6. A second filter on the two CIGAR was considered: only the lines with perfect CIGAR (xxSxxM) for both fragments were retained (the line 3 was not selected).

column	-1	-2	-3	-4	-5	-6	-7	-8	-9	-10	-11	-12	-13	-14	-15
line-1	18	41620104	+	18	41623937	+	0	0	1	HISEQ:29:C6l	41620061	43M82S	41623938	43S82M	*R2*
line-2	18	41620104	+	18	41623937	+	0	0	1	HISEQ:29:C6l	41620061	43M82S	41623938	43S82M	*R2*
line-4	18	55382707	+	18	55373242	+	1	2	0	HISEQ:29:C6l	55382649	58M67S	55373243	58S67M	*R2*
line-5	18	55382707	+	18	55373242	+	1	2	0	HISEQ:29:C6l	55382643	64M61S	55373243	64S61M	*R2*
line-6	18	55373242	-	18	55382707	-	2	0	2	D3VDZHS1:1!	55373243	60S40M	55382647	60M40S	*R1*
line-7	18	55373242	-	18	55382707	-	2	0	2	D3VDZHS1:1!	55373243	31S69M	55382676	31M69S	*R1*
line-8	18	55540797	-	18	55541187	-	0	0	0	D3VDZHS1:1!	55540798	66S34M	55541121	66M34S	*R1*
line-9	18	55540797	-	18	55541187	-	0	0	0	HISEQ:29:C6l	55540798	91S34M	55541096	91M34S	*R1*

To build the new table, we keep only columns -1, -2, -5, -6, -10 to -15.

column	c1	c2	c3	c4	c5	c6	c7	c8	c9	c10	c11
line-1	18	41620104	41623937	+	0	HISEQ:29:C6l	41620061	43M82S	41623938	43S82M	*R2*
line-2	18	41620104	41623937	+	0	HISEQ:29:C6l	41620061	43M82S	41623938	43S82M	*R2*
line-4	18	55382707	55373242	+	1	HISEQ:29:C6l	55382649	58M67S	55373243	58S67M	*R2*
line-5	18	55382707	55373242	+	1	HISEQ:29:C6l	55382643	64M61S	55373243	64S61M	*R2*
line-6	18	55373242	55382707	-	2	D3VDZHS1:1!	55373243	60S40M	55382647	60M40S	*R1*
line-7	18	55373242	55382707	-	2	D3VDZHS1:1!	55373243	31S69M	55382676	31M69S	*R1*
line-8	18	55540797	55541187	-	0	D3VDZHS1:1!	55540798	66S34M	55541121	66M34S	*R1*
line-9	18	55540797	55541187	-	0	HISEQ:29:C6l	55540798	91S34M	55541096	91M34S	*R1*

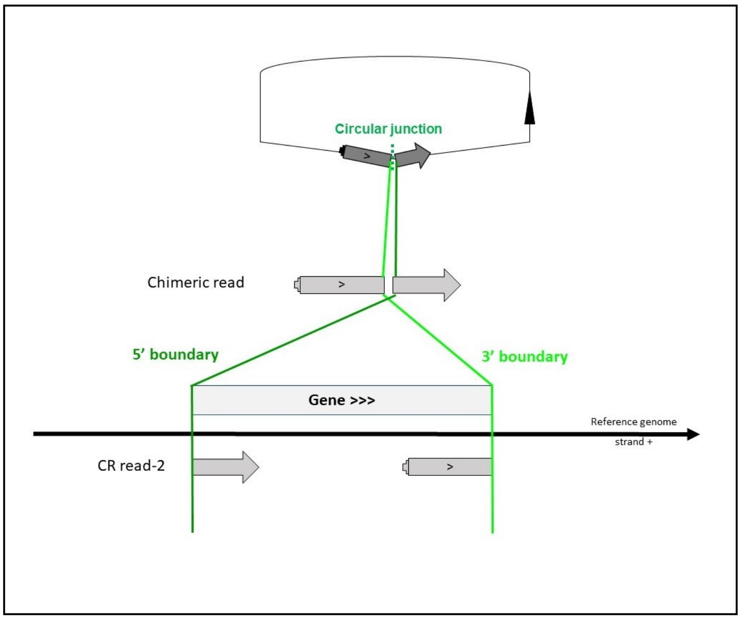

We propose to keep in c2 and c3, the genomic coordinates (inversion of c2 and c3 when c3<c2) defined with the boundaries of this mapping (correction of coordinates: new_c2=c2 +1 and new_c3=c3-1 corresponding to *1-based* coordinates of the circular transcript, as used in *gtf* files).

column	c1	C2	C3	c4	c5	c6	c7	c8	c9	c10	c11
line-1	18	41620105	41623936	+	0	HISEQ:29:C6U	41620061	43M82S	41623938	43S82M	*R2*
line-2	18	41620105	41623936	+	0	HISEQ:29:C6U	41620061	43M82S	41623938	43S82M	*R2*
line-4	18	55373243	55382706	+	1	HISEQ:29:C6U	55382649	58M67S	55373243	58S67M	*R2*
line-5	18	55373243	55382706	+	1	HISEQ:29:C6U	55382643	64M61S	55373243	64S61M	*R2*
line-6	18	55373243	55382706	-	2	D3VDZHS1:15	55373243	60S40M	55382647	60M40S	*R1*
line-7	18	55373243	55382706	-	2	D3VDZHS1:15	55373243	31S69M	55382676	31M69S	*R1*
line-8	18	55540798	55541186	-	0	D3VDZHS1:15	55540798	66S34M	55541121	66M34S	*R1*
line-9	18	55540798	55541186	-	0	HISEQ:29:C6U	55540798	91S34M	55541096	91M34S	*R1*

In this new file, c1-c4 rows contain the genomic coordinates of the LAC defined by the CR. **Now, we propose to select CR with both segments mapped in inverted order:**

From all reads, we select lines respecting two criteria :
When c4= « + » we retained lines if c7-c9>0 and c2=c9
When c4= « - » we retained lines if c7-c9<0 and c2=c7

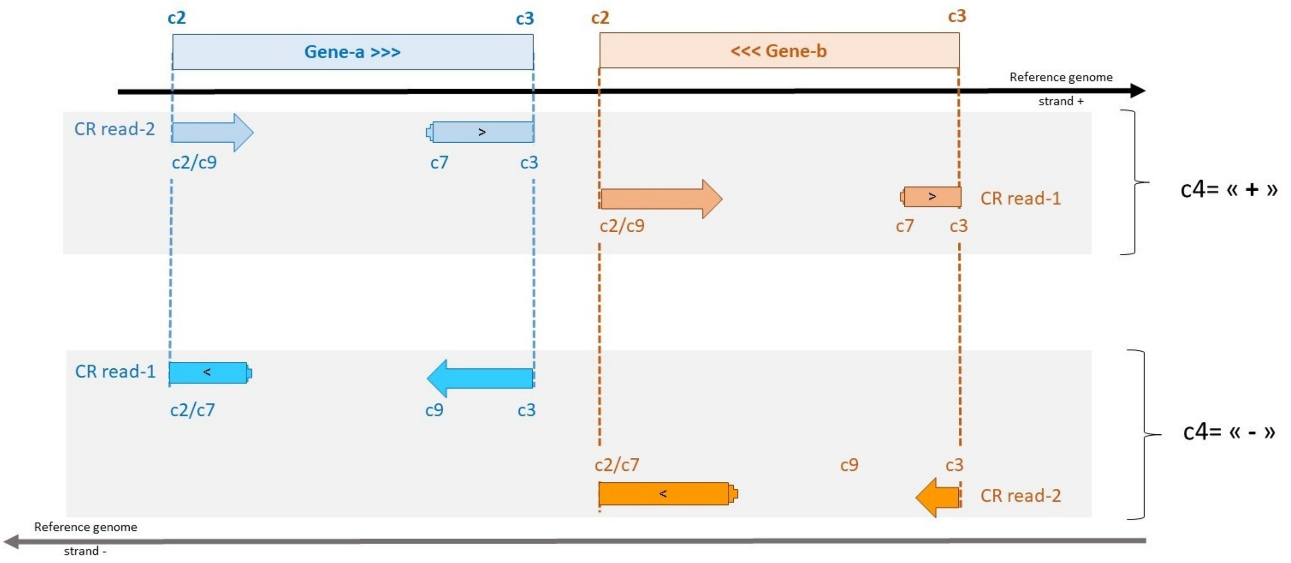

For this example, we chose two genes: the gene-a (in blue) is on the strand + of the reference genome and the gene-b (in brown) is located on the strand – of the reference genome.

When we applied this filter, line-1 and line-2 were discarded.

column	c1	C2	C3	c4	c5	c6	c7	c8	c9	c10	c11
line-4	18	55373243	55382706	+	1	HISEQ:29:C6U	55382649	58M67S	55373243	58S67M	*R2*
line-5	18	55373243	55382706	+	1	HISEQ:29:C6U	55382643	64M61S	55373243	64S61M	*R2*
line-6	18	55373243	55382706	-	2	D3VDZHS1:15	55373243	60S40M	55382647	60M40S	*R1*
line-7	18	55373243	55382706	-	2	D3VDZHS1:15	55373243	31S69M	55382676	31M69S	*R1*
line-8	18	55540798	55541186	-	0	D3VDZHS1:15	55540798	66S34M	55541121	66M34S	*R1*
line-9	18	55540798	55541186	-	0	HISEQ:29:C6U	55540798	91S34M	55541096	91M34S	*R1*

Last modifications

When the read is a read-1 the alignment is done on the opposite strand, consequently we must change the strand (c4) to define the same LAC. In fact we known only two genomic positions

The STAR classification concerning the splice junction (0= non canonical intronic motif, 1=GT/AG, 2=CT/AC) is done for the strand of the alignment. When the read is an R2 the canonical intronic motif is GT/AG and when the read is an R1 the canonical intronic motif is CT/AC.

At this step, we obtained the outfile CCR-file1.out.

The four columns of the CCR-file1.out contain the genomic coordinates defined by each CCR retained and we have still one line=one CCR. We used these four columns to group and count lines (CCR will be clustered) defining unique LACs.

CCR-file1.out It is the out file of the process of selection of chimeric reads mapped in inverted order and it is a tabular file containing the list of all CCRs. This file contains the genomic coordinates of the LAC defined by the CR (c1, c2, c3 and C4). We kept the name of the read in c6, the type of read (R1 or R2 in c11) and the STAR classification concerning the splice junction (no= absence of canonical intronic motif, yes= presence of canonical intronic motif) in C5. In c7-c11, we kept the characteristic of the CCR and these columns will be useful to characterize "distinct CCR".

column	c1	C2	C3	**C4**	**C5**	c6	c7	c8	c9	c10	c11
line-4	18	55373243	55382706	+	yes	HISEQ:29:C6l	55382649	58M67S	55373243	58S67M	*R2*
line-5	18	55373243	55382706	+	yes	HISEQ:29:C6l	55382643	64M61S	55373243	64S61M	*R2*
line-6	18	55373243	55382706	+	yes	D3VDZHS1:1!	55373243	60S40M	55382647	60M40S	*R1*
line-7	18	55373243	55382706	+	yes	D3VDZHS1:1!	55373243	31S69M	55382676	31M69S	*R1*
line-8	18	55540798	55541186	+	no	D3VDZHS1:1!	55540798	66S34M	55541121	66M34S	*R1*
line-9	18	55540798	55541186	+	no	HISEQ:29:C6l	55540798	91S34M	55541096	91M34S	*R1*

CCR-file2.out It is the outfile of the grouping process: CCR are clustered by the LAC that they define. This file contains the columns c1, c2, c3 and C4 from the CR-file1.out (genomic coordinates) and a new column (#5) including the number of CR identified for the LAC considered (the number of lines counted).

column	c1	C2	C3	C4	#5
	18	55373243	55382706	+	25
	18	55540798	55541186	+	2

The output file of the process of selecting chimeric reads mapped in inverted order is a tabular file (CCR-file1.out) containing the list of all CCRs. The four columns contain the genomic coordinates defined by each CCR retained and we have one line per CCR retained. To perform the clustering of CCRs, we used the four first columns to group and count lines defining unique LACs. This new output file (CCR-file2.out) contains the genomic coordinates and a new column including the number of CCR found for the LAC considered. After the application of the threshold concerning the number of CCR retained by LAC, we obtained the CCR-file3.out.

This file contains also coordinates of each LAC defined by the corresponding CCR (chr, start, end, and strand) and it is suitable for bed intersect process. In fact, we known only the two borders of this LAC, which are the two points involved in the back and circular junction. The back junction was codified on three columns (5′ boundary [chr:start

strand], 3′ boundary [chr:start strand], and the name of this back junction [chr:start-end strand]) and these three columns allows comparative analyses with an exons-file.

CCR-file3.out it is the out file obtained after applying a threshold on the number of CCR retained by LAC.
CJ= Back junction

Chromosome	Start	End	Strand	N. of CR	Genomic size	5' boudary of the CJ	3' boudary of the CJ	Name of the Circular Junction
18	55373243	55382706	+	25	9464	18:55373243+	18:55382706+	18:55373243-55382706+
12	14867960	14868030	-	70	71	12:14867960-	12:14868030-	12:14867960-14868030-
13	120756800	120779879	+	45	23080	13:120756800+	13:120779879+	13:120756800-120779879+
10	45992309	46032747	+	5	40439	10:45992309+	10:46032747+	10:45992309-46032747+
1	608276	611340	+	57	3065	1:608276+	1:611340+	1:608276-611340+
1	608276	619011	+	27	10736	1:608276+	1:619011+	1:608276-619011+
10	45992309	46027671	+	62	35363	10:45992309+	10:46027671+	10:45992309-46027671+
10	46002429	46022353	+	11	19925	10:46002429+	10:46022353+	10:46002429-46022353+
13	118870848	118874998	+	7	4151	13:118870848+	13:118874998+	13:118870848-118874998+
13	120745752	120779879	+	5	34128	13:120745752+	13:120779879+	13:120745752-120779879+
12	14867963	14868033	-	84	71	12:14867963-	12:14868033-	12:14867963-14868033-
10	46002429	46006364	+	7	3936	10:46002429+	10:46006364+	10:46002429-46006364+
13	120756800	120765156	+	60	8357	13:120756800+	13:120765156+	13:120756800-120765156+

This file contains the coordinates of each LAC on four columns (and it is suitable for bed intersect process). In fact, we known only the two borders of this LAC, which are the two points involved in the circular junction. The circular junction was codified on three columns (5′ boundary [chr:startStrand], 3′ boundary [chr:start Strand], and the name of this circular junction [chr:start-end strand]). These columns allow comparative analyses with an exons-file.
This file contains also two columns reporting the number of CCRs found for the LAC/circular junction considered and the genomic distance between the two borders of the LAC defined by CCR.

In the CCR-file1.out we kept the name of the read and the information on the circular junction given by STAR mapping (presence or absence of canonical GT/AG splicing signal at circRNA junction borders). From the file CCR-file3.out we can go back to the list of CCRs of a specific region (CCR-file1.out) to analyze the STAR splice junction classification of the splice site. Moreover, we can use this list of CCRs to obtain their sequences and sequences of their mate-pair. Only the 18 autosomes from porcine genome have been considered in this study.

All analyses using tabular files were managed on the Galaxy platform from Toulouse http://bioinfo.genotoul.fr/

3. Annotation of exonic circRNAs

3.1. Ensembl

The complete porcine annotation proposed by *Ensembl* for the porcine specie was analyzed (release 97) to extract several lists of exons. The annotation of porcine genome makes the distinction between coding and non-coding transcripts, but also never associates a nc transcript with a coding transcript in the same gene.

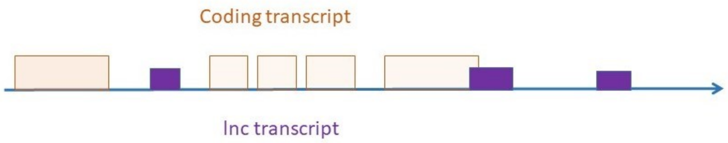

release 97			
	**N. of exons**		**size of the exon**
**gene_biotype "protein_coding"**	**521,056**	1,868/21,074 mono-exonic genes	
**gene_biotype "pseudogene"**	**288**	0/126 mono-exonic gene	
**gene_biotype "linc"**	**1,745**	0/316 mono-exonic gene	
**gene_biotype "IG_C_gene"**	**1**	1/1 mono-exonic gene	317 bp
**gene_biotype "IG_V_gene"**	**10**	8/9 mono-exonic gene	269-299 bp
**gene_biotype "miRNA"**	**484**	484/484 mono-exonic gene	52-144 bp
**gene_biotype "misc_RNA"**	**379**	379/379 mono-exonic gene	31-471 bp
**gene_biotype "ribozyme"**	**9**	9/9 mono-exonic gene	77-365 bp
**gene_biotype "rRNA"**	**190**	190/190 mono-exonic gene	34-149 bp
**gene_biotype "scaRNA"**	**29**	29/29 mono-exonic gene	81-433 bp
**gene_biotype "snoRNA"**	**588**	588/588 mono-exonic gene	38-328 bp
**gene_biotype "snRNA"**	**1,044**	1,044/1,044 mono-exonic gene	50-198 bp
**gene_biotype "sRNA"**	**3**	3/3 mono-exonic gene	57-82 bp
**gene_biotype "TR_J_gene"**	**3**	3/3 mono-exonic gene	59-62 bp
**gene_biotype "TR_V_gene"**	**70**	70/70 mono-exonic gene	35-302 bp

3.2. ALDB database

A lot of lnc transcripts have been characterized in pig but the genomic coordinates of each exon are only available for those available in the *ALDB* database (domestic-Animal Long noncoding RNA DataBase, [3]).

http://202.200.112.245/aldb/

This database reports more than 15,000 lnc but coordinates are given for Sscrofa10.2. Nevertheless, these lnc are described including the genomic coordinates of each exon. We used the UCSC-tool (https://genome.ucsc.edu/cgi-

bin/hgLiftOver) to convert the coordinates of each exon. As the strand of the region was not considered, we used the converted exons with both possible strands.

3.3. Creation of list of novel exons

From the three double datasets (total-RNA-seq and mRNA-seq available for three samples), we elaborated a strategy to identify a maximum of novel exons. Our purpose is not to provide a list of new annotated exons. We are aware of the small number of data sets and the difficulties of such characterization.
The three animals were 31, 05 and 54 previously described [1].

To characterize new exons, reads from Total-RNA-seq and from mRNA-seq were mapped with HISAT2 [4]. Within the Total RNA-seq and mRNA-seq data separately, Cufflinks [5] were used on each of the three Bam files and on the merged Bam file to define new transcripts. Two sets of four files "assembled transcripts" were obtained (*gtf* files).

Cufflinks X4<br>mRNA-seq	Cufflinks X4<br>Total-RNA-seq

In a first way (represented at the bottom of the diagram), we recovered news exons included in these 2X4 files. We noted the loss of exons with an undefined strand.

In a second way (represented at the top of the diagram), we used Cuffcompare [5] with these two sets of "assembled transcripts" files. The option "Discard single-exon transfrags and reference transcripts » was actived. We noted the loss of exons without a validate links with another exon.

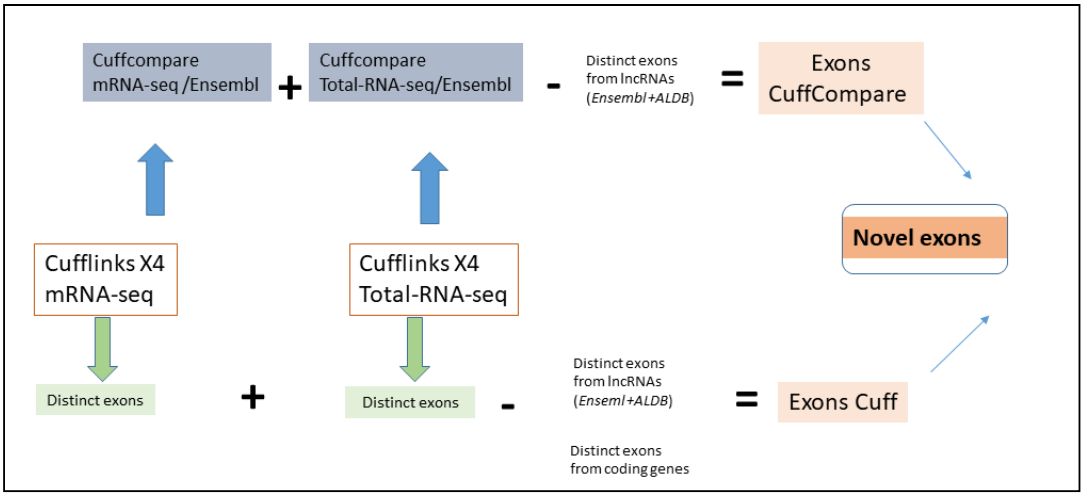

3.4. *Exons files and uses*

All initial files containing coordinates of exons are *gtf* files (*1-based)*.
Each exons-file contains the coordinates of each LAC on four columns (and it is suitable for *bed intersect* process). Moreover each exon was codified on three columns (5' exonic boundary [chr:start strand], 3'exonic boundary [chr:start strand], and name of the exon [chr:start-end strand]). These columns allow comparative analyses with a CCR-file3.out file.

To identify exonic circRNAs, we propose to compare the boundaries of LACs defined by each CCR to boundaries of described exons. Only the exact matches are considered.

column	c1	c2	c3	C4	#5	#6	5' boundary of the circular junction	3' boundary of the circular junction	Name of the circular junction
	18	55373243	55382706	+	25	9464	**18:55373243+**	**18:55382706+**	18:55373243-55382706+
	18	55540798	55541186	+	2	389	18:55540798+	18:55541186+	18:55540798-55541186+

In this example, only the LAC reported in blue can be annotated as exonic circRNA.

				**exons-file_Ensembl coding genes**		
				5' Exonic boundary	3' Exonic boundary	name of the exon
18	55271586	55271663	+	18:55271586+	18:55271663+	18:55271586-55271663+
18	55271681	55271860	+	18:55271681+	18:55271860+	18:55271681-55271860+
18	55301604	55301660	+	18:55301604+	18:55301660+	18:55301604-55301660+
18	55301635	55301660	+	18:55301635+	18:55301660+	18:55301635-55301660+
18	55312687	55312725	+	18:55312687+	18:55312725+	18:55312687-55312725+
18	55342610	55342717	+	18:55342610+	18:55342717+	18:55342610-55342717+
18	55350052	55350129	+	18:55350052+	18:55350129+	18:55350052-55350129+
18	55373243	55373317	+	**18:55373243+**	18:55373317+	18:55373243-55373317+
18	55379632	55379694	+	18:55379632+	18:55379694+	18:55379632-55379694+
18	55382641	55382706	+	18:55382641+	**18:55382706+**	18:55382641-55382706+
18	55537411	55537537	+	18:55537411+	18:55537537+	18:55537411-55537537+
18	55538733	55538825	+	18:55538733+	18:55538825+	18:55538733-55538825+
18	55539876	55544699	+	18:55539876+	18:55544699+	18:55539876-55544699+
18	55544886	55545047	+	18:55544886+	18:55545047+	18:55544886-55545047+
18	55548917	55549318	+	18:55548917+	18:55549318+	18:55548917-55549318+
18	55549404	55549530	+	18:55549404+	18:55549530+	18:55549404-55549530+
18	55549661	55549789	+	18:55549661+	18:55549789+	18:55549661-55549789+
18	55554650	55554778	+	18:55554650+	18:55554778+	18:55554650-55554778+

4. Annotation of intronic circRNA

As described by Zhang et al. [6] for intronic circRNAs, the first boundary of the circular junction has to coincide with the beginning of the intron (-5/+5 bp) and the second boundary must be compatible with a circularization event limited by the branch-point (we accepted a distance from the end of the intron of 15 to 32 nt). For intron circles, each boundary of the circular junction must coincide with the beginning and the end of the intron (-5/+5 bp).
Following the study of Zhang et al. (2013) [6], we decided to limit the selection of intron-derived circRNAs to those with < 5,000 bp.

1. Robic, A; Faraut, T; Djebali, S; Weikard, R; Feve, K; Maman, S; Kuehn, C: Analysis of pig transcriptomes suggests a global regulation mechanism enabling temporary bursts of circular RNAs. *RNA Biol* **2019**, *16*,1190-1204.
2. Cheng, J; Metge, F; Dieterich, C: Specific identification and quantification of circular RNAs from sequencing data. *Bioinformatics* **2016**, *32*,1094-1096.
3. Li, A; Zhang, J; Zhou, Z; Wang, L; Liu, Y; Liu, Y: ALDB: a domestic-animal long noncoding RNA database. *PLoS One* **2015**, *10*,e0124003.
4. Kim, D; Langmead, B; Salzberg, SL: HISAT: a fast spliced aligner with low memory requirements. *Nat Methods* **2015**, *12*,357-360.
5. Trapnell, C; Williams, BA; Pertea, G; Mortazavi, A; Kwan, G; van Baren, MJ; Salzberg, SL; Wold, BJ; Pachter, L: Transcript assembly and quantification by RNA-Seq reveals unannotated transcripts and isoform switching during cell differentiation. *Nat Biotechnol* **2010**, *28*,511-515.
6. Zhang, Y; Zhang, XO; Chen, T; Xiang, JF; Yin, QF; Xing, YH; Zhu, S; Yang, L; Chen, LL: Circular intronic long noncoding RNAs. *Mol Cell* **2013**, *51*,792-806.

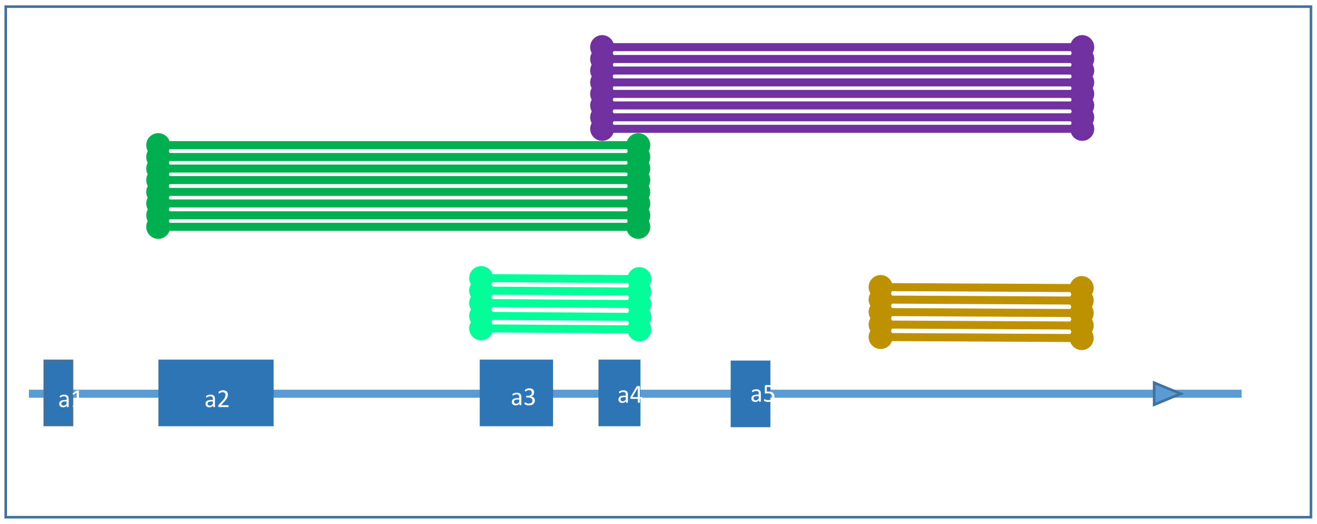

In this region containing a gene with 5 described exons, we observed four sets of CCRs.

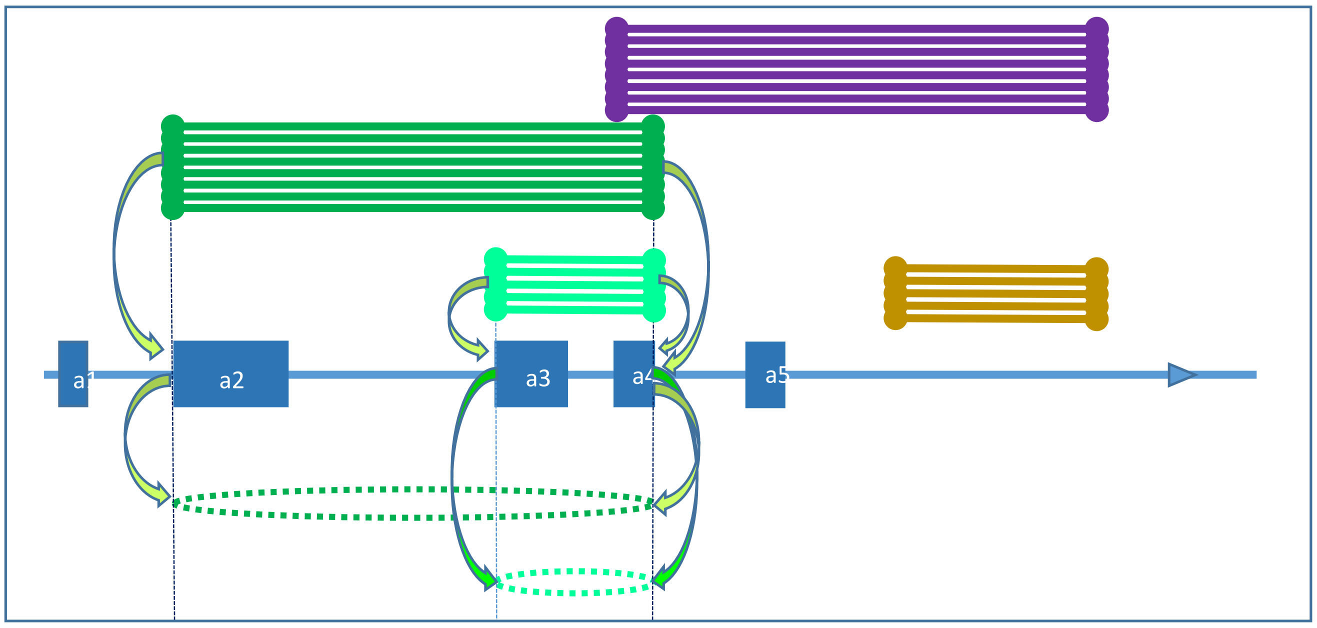

We start the process by identifying the genomic regions which have their borders (genomic coordinates) matching exactly to boundaries of described exons.

The both circRNAs drawn in green have their both borders which match exactly to boundaries of described exons.

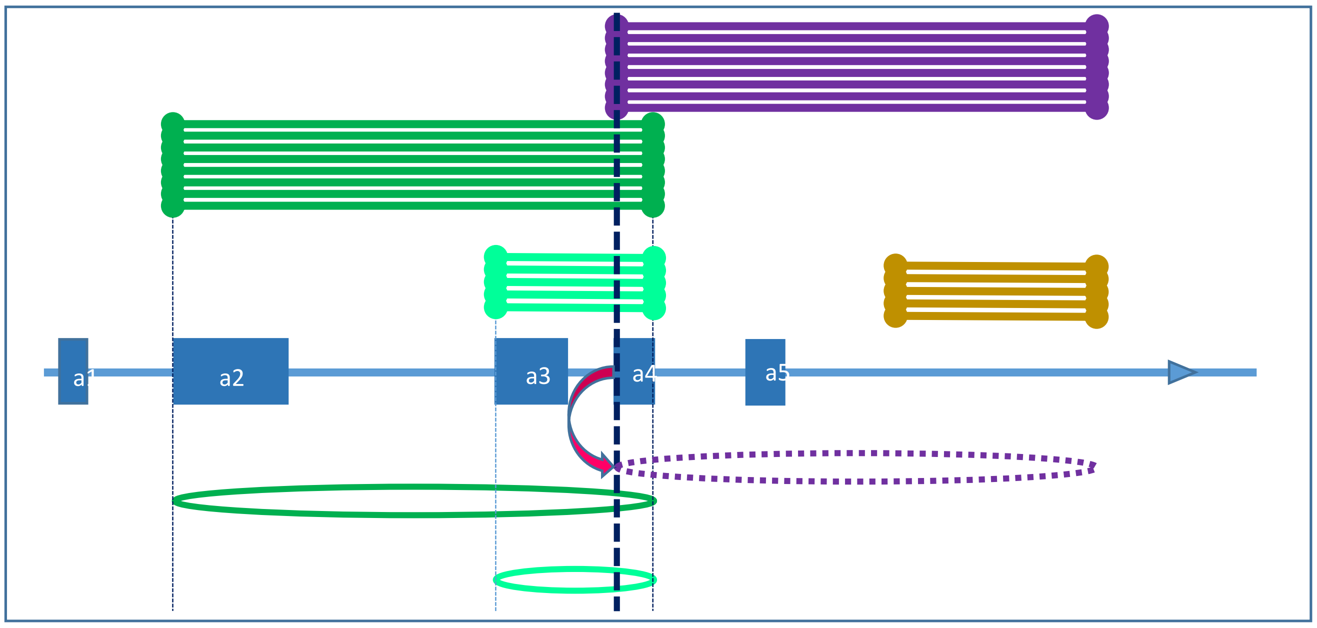

The circRNAs drawn in purple have only one border which matches exactly to a boundary of described exons.

After the first round, we identified some circRNAs with only one known boundary, We hypothesize that the second border of circRNAs are the limits of uncharacterized exons.

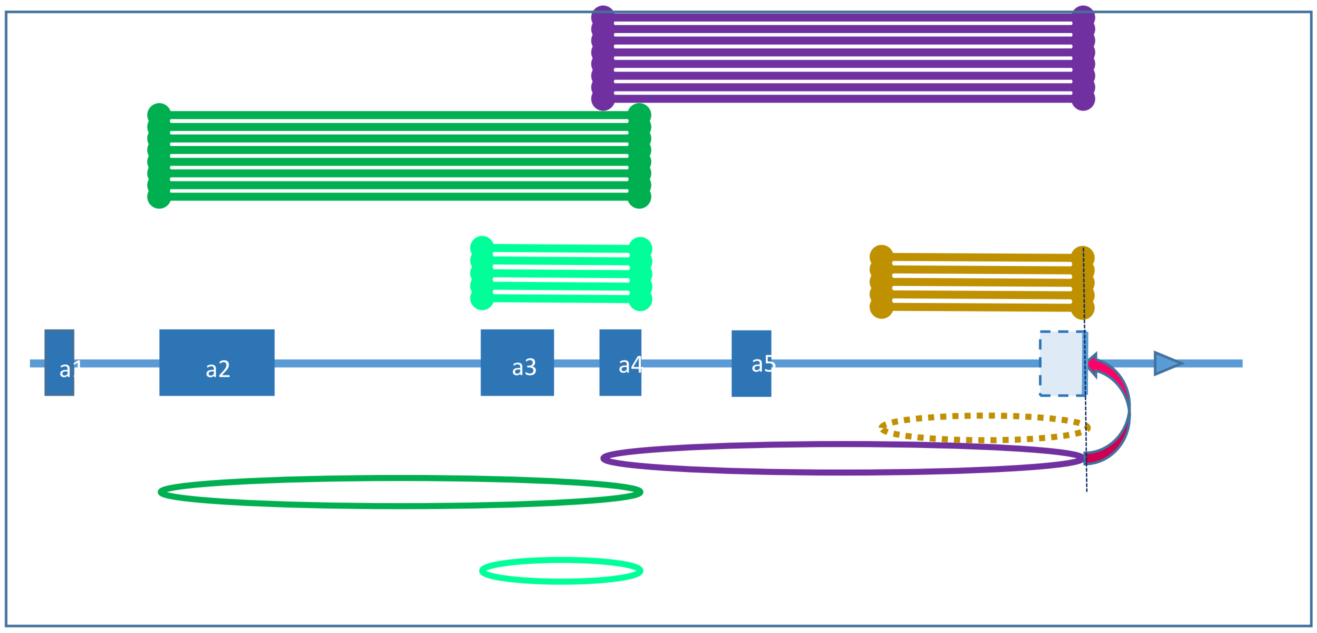

We propose to use these novel exonic boundaries in a second round of identification.

We repeat this process until there are no more answers.

Iterative strategy to identify all exonic circRNAs originating from a list of genes. The description of this strategy is illustrated with an example where we considered a gene, including three described exons and producing four circular RNAs. With a classical strategy, the annotation of the circRNA drawn in gold would be completely inaccessible.

Supplementary Document 2A

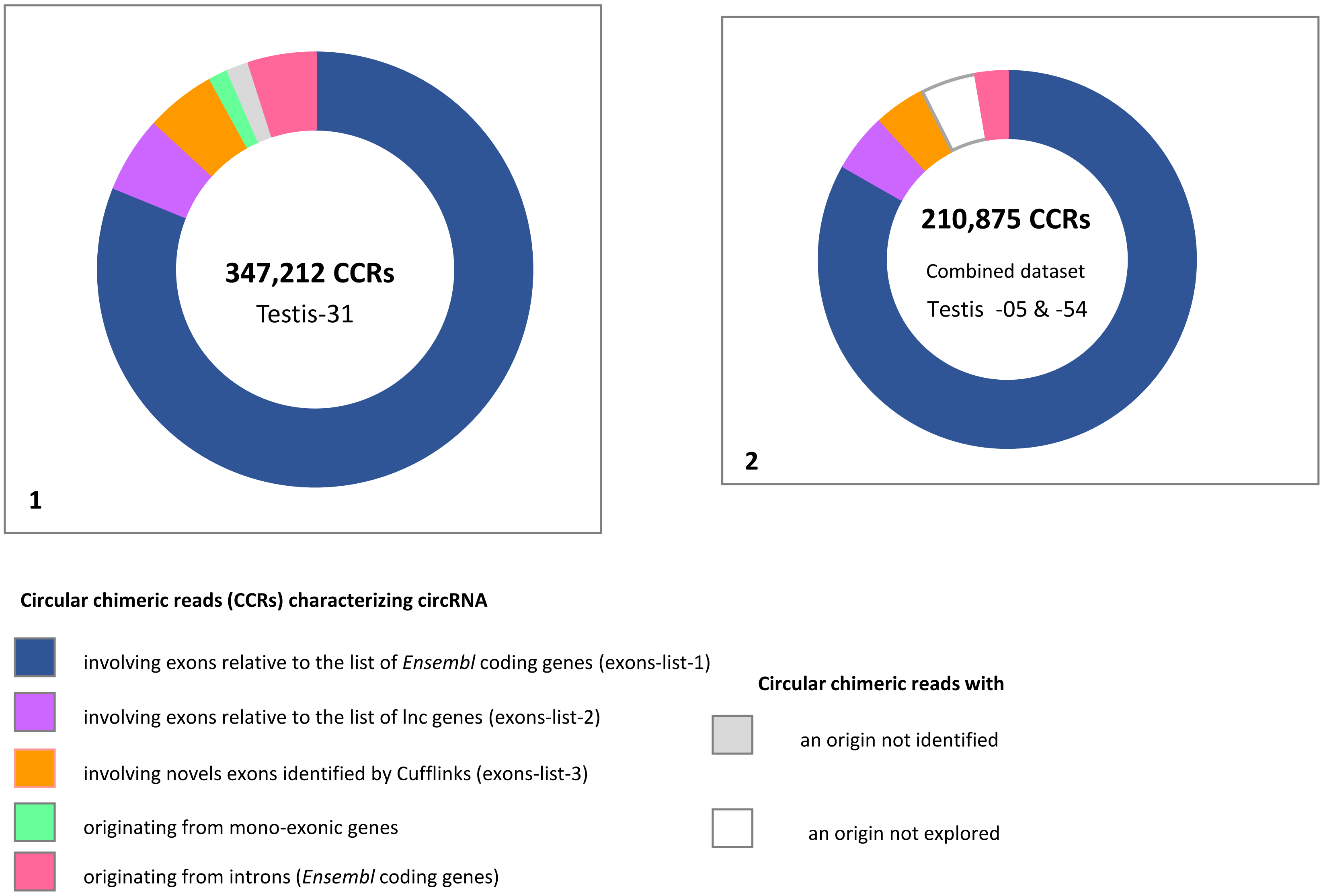

Genomic origin of Circular chimeric reads (CCRs) identified in the Testis-31 and in a combined dataset. (1) Testis-31: Identification of the genomic origin of the 347,212 CCRs. (2) Combined dataset (Testis-05 & -54): Identification of the origin of the 210,875 CCRs considered as coming from circRNAs.

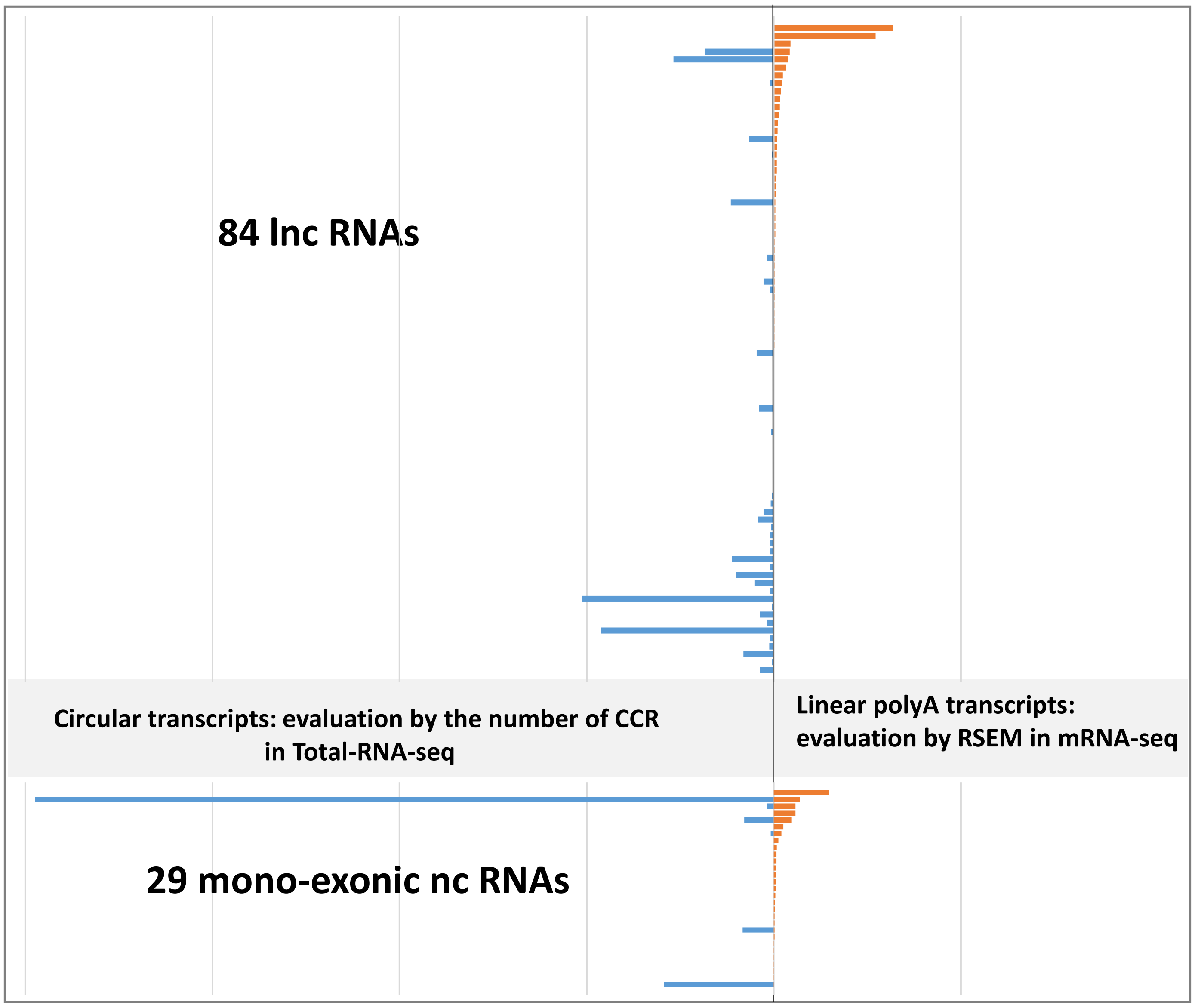

Absence of relationship between circRNA and mRNA productions from non-coding genes

84 lnc and 29 mono-exonic and short nc (exon>200 bp) genes were considered in this pairwise comparison.

Supplementary Document 2C

Supplementary document 3

3A **Reads examination**
3B **CircRNAs and mono-exonic genes**

3A Reads examination

A1 **Intronic circRNA(s) from the intron 21 of *ATXN2L* (ENSSSCG00000021845)**

A2 **Sub-exonic circRNA(s) of 145 nt. from *RNAse-MRP* locus (ENSSSCG00000018700)**
Five LACs. Two distinct sub-exonic circRNAs of 145 nt were validated.

A3 **Sub-exonic circRNA(s) of 115 nt. from *RNAse-MRP* locus (ENSSSCG00000018700)**
Two LACs. Only one sub-exonic circRNA, and it was validated

A4 **Sub-exonic circRNA(s) of 61 nt. from *RNAse-MRP* locus (ENSSSCG00000018700)**
We are not able to validate the hypothesis of a small circRNA.

A5 **circRNA(s) from the monoexonic gene ENSSSCG00000019194 (*SNORD104)***
Two LACs. Only one circRNA, and it was validated

We considered for the following analyses all CCRs retained for a sub-region suspected to produce several sub-exonic circRNAs (with the same size) or suspected to produce an intronic circRNA. For each CCR, the second mate of the pair was recovered and multiple alignments were performed. The sequence contents of these reads was examined to validate the circular structure. Moreover, we tried to characterize different circularization events.
For very small circRNA we could expect to find reads containing twice the circular junction, but these type of reads were not retained by STAR. Nevertheless, among the reads classified as CCR we can expect to find the circular junction in both mates of a pair.
For all circRNA, when we have a read, that is considered as CCR, the second mate of the pair must be compatible with a circular transcript.

A1 Intronic circRNA(s) from the intron 21 of *ATXN2L* (ENSSSCG00000021845)

ATXN2L (Pig Gene, Strain: reference)
ENSSSCG00000021845 3:18506052-18519060:1

>seq of the intron

GTAGGAGCCCACTCCACCCGAACGGGGGGCTGCCCCATCCCGCATCCTTGGGCACCTCTTCACTCTGCTCCTGACGCTAGGATCCGC
AGAGCCCGCGACAAGCTCCCGTGGTGCTCCCAGCTCCTTTTATTTGTTCTTTGCAG

11,857 CCR (2,511 Reads-1 and 9,346 Reads-2) characterizing the intronic circRNAs originating from this intron of *ATXN2L* lead defining 10 LACs. We studied the sequence of these 9,895 PE.

After the examination of several multiple alignments, we observed variations of sequences at the circular junction (symbolized by |).

In the order of best frequency

GACAAGCTCCCGTGGTGCTC | GTAGGAGCCCACTCCACCC

GACAAGCTCCCGTGGTGCTCC | GTAGGAGCCCACTCCACCC

GACAAGCTCCCGTGGTGC | GTAGGAGCCCACTCCACCC

GACAAGCTCCCGTGGTGCT | GTAGGAGCCCACTCCACCC

>7882R1
CGACAAGCTCCCGTGGTGC|GTAGGATCCCACTCCACCCGATCGGGGGGCTGCCCCATCCCGCATCCTTGGGCACCTCTTCACTC
TGCTCCTGACGCTAGGATCCGCAGAGCCCGCGACAAGCTCC

>7582R2
CGCTAGGATCCGCAGAGCCCGCGACAAGCTCCCGTGGTGCTC|GTAGGAGCCCACTCCACCCGAACGGGGGGCTGCCCCATCCC
GCATCCTTGGGCACCTCTTCACTCTGCTCCTGACGCTAGGAT

>9910R1
GCCCGCGACAAGCGCCCGCGGTGCTC|GTAGGAGCCCACTCCACCCGAA*CCGGGCGCTGCCCCATCCCG*CATCCTTGGGCACCTC
TTCACTCTGCTCCTGACGCTAGGATCCGCAGAGCCCGCGAC
>9910R2
CCTTGGGCACCTCTTCACTCTGCTCCTGACGCTAGGATCCGCAGAGCCCGCGACAAGCTCCCGTGGTGCTC|GTAGGAGCCCACT
CCACCCGAACGGGGGGCTGCCCCATCCCGCATCCTTGGGCA

>8004R1
ACAAGCTCCCGTGCTGC|GTAGGAGCCCACTCCACCCGAACGGGGGGCTGCCCCATCCCGC*ATCCTTGGG*CACCTCTTCACTCTG
CTCCTGACGCTAGGATCCGCAGAGCCCGCGACAAGCTCCCG
>8004R2
CCACTCCACCCGAACGGGGGGCTGCCCCATCCCGCATCCTTGGGCACCTCTTCACTCTGCTCCTGACGCTAGGATCCGCAGAGCC
CGCGACAAGCTCCCGTGGTCC|GTAGGA-CCCCGCTCCGCCC

>8005R1
CCCGTGGTGCTC|GCAGGAGCCCACTCCACCCGACCGGGGGGCTGCCCCATCCCGCATCCTTGGGCACCTCTTCACTCTGCTCCT
GACGCTAGGATCCGCAGAGCCCGCGACAAGCTCCCGTGGCG
>8005R2
GCCCCATCCCGCATCCTTGGGCACCTCTTCACTCTGCTCCTGACGCTAGGATCCGCAGAGCCCGCGACAAGCTCCCGTGGTGC|TC
GTAGGAGCCCACTCCACCCGAACGGGGGGCTGCTCCCTCC

>8007R1
GCGACAAGCTCCCTTGGTGCTC|GTAGGAGCCCACTCCCCCCGAACGGGGGGCTGCCCCATCCCGCATCCTTGGGCACCTCTTCA
CTCTGCTCCTGACGCTAGGATCCGCAGAGCCCGCGACAAGC
>8007R2
CGGGGGGCTGCCCCATCCCGCATCCTTGGGCACCTCTTCACTCTGCTCCTGACGCTAGGATCCGCAGAGCCCGCGACAAGCTCCC
GTGGTGCTC|GTAGGAGCCCACTCCACCCGAACTGTGGGCT

>8042R1
CTCCCGTGGTGCTC|GTAGGAGCCCACTCCCCCCGAACGGGGGGCTGCCCCATCCCGCCTCCTTGGGCACCTCTTCACTCTGCTCC
TGACGCTAGGATCCGCAGAGCCCGCGACAAGCTCCCGTGG
>8042R2
CCTTGGGCACCTCTTCACTCTGCTCCTGACGCTAGGATCCGCAGAGCCCGCGACAAGCTCCCGTGGTGCTC|GTAGGAGCCCACT
CCACCCGAACGGGGGGCTGCCCCATCCCGCCTCCTTGGGCA

This analysis confirmed difficulties (lower fidelity) for reverse transcriptase near the 2’-5’ link. Moreover, the polymerase used in sequencing has also a lower fidelity in GC rich regions, and here sequences located downstream of this back junction were impacted. Without this problem of unreliable sequencing in this intron, we could expect a higher number of CCR. This example shows the interest to perform the mapping in single-end process and not discard systematically the CCR when the second mate is not concordant with the mapping of the read that is chimeric.

This analysis validates that this intron (intron 21 of *ATXN2*L) is able to produce circRNAs.

A2 Sub-exonic circRNA(s) of 145 nt. from RNase-MRP locus (ENSSSCG00000018700)

RNase_MRP (Pig Gene, Strain: reference)
ENSSSCG00000018700 1:236380019-236380276:-1
ENSSSCT00000020295.2

227 CCRs were mapped by STAR on the LAC [1:236380021-236380165-] and they are only Read-1.
15 CCRs were mapped by STAR on the LAC [1:236380026-236380170-] and they are only Read-2.
7 CCRs were mapped by STAR on the LAC [1:236380028-236380172-] and they are only Read-2.
94 CCRs were mapped by STAR on the LAC [1:236380027-236380174-] and they are only Read-1.
49 CCRs were mapped by STAR on the LAC [1:236380029-236380173-] (42 Read-1 & 7 Read-2).

We considered 374 PE for this region.
735/748 reads are compatible with a transcript, which would be circular. In addition, we found 13 Reads-2 containing sequence localized outside of the borders of these circRNAs.

>the single exon of *RNAse_MRP*
GCTCGTGCTGAAGGCCTGTTTCCTAGGCTACAAACGAGGGACTAGTTCCTTATTTACGCCTAGGGGAAAGTCCCCGGACCTAAGGCA
GAGAGTGCCACGTGCGCCTGCACGTAGACTGTCCCTGCACCTCACGGTTAATCCGCTAAGAAGCGATTTCGCCGAGCGGCGTGTGGC
AGGGATGTCATCCGTCAGCCGAAATAGTTACACAGGCAGTGCGCTTCCGCGCACCAACCACACGGGGCTCATTCTCAGCACGGC

Wth these 374 PE, we are able to suggest two consensus sequences including two possible back-junctions (green| greyyellow and greengrey | yellow). These both consensus sequences identified two different circRNAs because they are originating from two distinct circularization events.

ACACGGGGCTCATTCTC| TGCACGTAGACTGTCCCTGCACCTCACGGTTA
ACACGGGGCTCATTCTCAGCACG |TAGACTGTCCCTGCACCTCACGGTTA

>R1-298
AGGCAGTGCGCTTCCGCGCACCAACCACACGGGGCTCATTCTCTGCACGTAGACTGTCCCTGCACCTCACGGTTAATCCGCTAAGAAGCGATTT
CGCCGAGCGGCGTGTGGCGGGGATGTCATCC
>298
CATCCGTCAGCCGAAATAGTTACACAGGCAGTGCGCTTCCGCGCACCAACCACACGGGGCTCATTCTCTGCACGTAGACTGTCCCTGCACCTCA
CGGTTAATCCGCTAAGAAGCGATTTCGCCGA

First circular RNA
CCGAAATAGTTACACAGGCAGTGCGCTTCCGCGCACCAACCACACGGGGCTCATTCTC|TGCACGTAGACTGTCCCTGCACCTCACG
GTTAATCCGCTAAGAAGCGATTTCGCCGAGCGGCGTGTGGCAGGGATGTCATCCGTCAG

>R1-82
GGCAGTGCGCTTCCGCGCACCAACCACACGGGGCTCATTCTCAGCACGTAGACTGTCCCTGCACCTCACGGTTAATCCG
CTAAGAAGCGATTTCGCCGAGCGGCGTGTGGAAGGGATGTCATCCG
>R2-82
CACAGGCAGTGCGCTTCCGCGCACCAACCACACGGGGCTCATTCTCAGCACGTAGACTGTCCCTGCACCTCACGGTTAA
TCCGCTAAGAAGCGATTTCGCCGAGCGGCGTGTGGAAGGGATGTCA
Second circular RNA

TAGTTACACAGGCAGTGCGCTTCCGCGCACCAACCACACGGGGCTCATTCTCAGCACG|TAGACTGTCCCTGCACCTCACGGTTAAT
CCGCTAAGAAGCGATTTCGCCGAGCGGCGTGTGGCAGGGATGTCATCCGTCAGCCGAAA

This analysis leads us to propose only two distinct sub-exonic circRNAs (145 nt)

A3 Sub-exonic circRNA(s) of 115 nt. from RNase-MRP locus (ENSSSCG00000018700)

RNase_MRP (Pig Gene, Strain: reference)
ENSSSCG00000018700 1:236380019-236380276:-1
ENSSSCT00000020295.2

99 CCR were identified for the LAC [1:236380021-236380135-], all were Reads-2.
230 CCR were identified for the LAC [1:236380018-236380132-], all were Reads-1.
The both reads are chimeric for 91 PE (the read 1 mapped on the LAC [1:236380018-236380132-] and the read-2 mapped [1:236380021-236380135-]). With these 238 PE, we are able to suggest a unique consensus sequence

including the back-junction. Among Reads-1 and among Reads-2 considered, we found 238/238 and 201/238 including the back junction (pinkgrey | blue). We did not find any read discordant with this suggested circRNA.

GCTCATTCTCAGCACGGC | TAAGAAGCGATTTCGCCGAGC

>the single exon of *RNAse_MRP*

GCTCGTGCTGAAGGCCTGTTTCCTAGGCTACAAACGAGGGACTAGTTCCTTATTTACGCCTAGGGGAAAGTCCCCGGACCTAAGGCA
GAGAGTGCCACGTGCGCCTGCACGTAGACTGTCCCTGCACCTCACGGTTAATCCGCTAAGAAGCGATTTCGCCGAGCGGCGTGTGGC
AGGGATGTCATCCGTCAGCCGAAATAGTTACACAGGCAGTGCGCTTCCGCGCACCAACCACACGGGGCTCATTCTCAGCACGGC

>Seq consensus R1

GGGATGTCATCCGTCAGCCGAAATAGTTACACAGGCAGTGCGCTTCCGCGCACCAACCACACGGGGCTCATTCTCAGCACGGCTAAG
AAGCGATTTCGCCGAGCGGCGTGTGGCGGGGATGTCATCC

>Seq consensus R2

CGCCGAGCGGCGTGTGGCGGGGATGTCATCCGTCAGCCGAAATAGTTACACAGGCAGTGCGCTTCCGCGCACCAACCACACGGG
GCTCATTCTCAGCACGGCTAAGAAGCGATTTCGCCGAGCGGCGTGTGGCGGGGATGTC

Example of PE

>R1-88

GGATGTCATCCGTCAGCCGAAATAGTTACACAGGCAGTGCGCTTCCGCGCACCAACCACACGGGGCTCATTCTCAGCACGGCTA
AGAAGCGATTTCGCCGAGCGGCGTGTGGAAGGGATGTCATC

>R2-88

CGCTAAGAAGCGATTTCGCCGAGCGGCGTGTGGAAGGGATGTCATCCGTCAGCCGAAATAGTTACACAGGCAGTGCGCTTCCGC
GCACCAACCACACGGGGCTCATTCTCAGCACGGCTAAGAAG

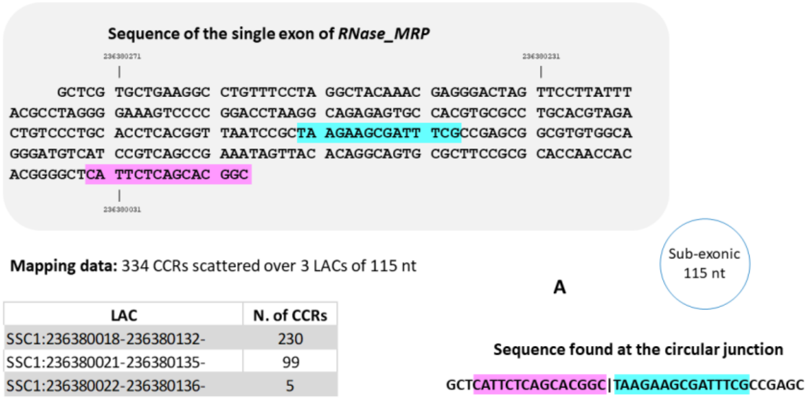

All reads examined were compatible with a unique sub-exonic circRNA including 115 nt

Suppl. Doc. 3A

A4 Sub-exonic circRNA(s) of 61 nt. from RNase-MRP locus (ENSSSCG00000018700)

RNase_MRP (Pig Gene, Strain: reference)
ENSSSCG00000018700 1:236380019-236380276:-1
ENSSSCT00000020295.2

Two LACs were identified by the selection of CCR mapped in inverted order.

12 CCRs were identified for the LAC [1:236380035-236380095-], all were Reads-2.

74 CCRs were identified for the LAC [1:236380031-236380091-], all were Reads-1.

Among Reads-1 and among Reads-2, we found 77/77 and 21/77 including a back junction (pink | green) but always once while we have a large part of reads of 125 bp. Among Reads-1 and among Reads-2, we found 86/144 including sequences not expected if we have a circRNA of 61 nt.

For example, three pairs of reads

>874-R2
CGCTAAGAAGCGATTTCGCCGAGCGGCGTGTGGAAGGGATGTCATCCGTCAGCCGAAATAGTTACACAGGCAGTGCGCTTCCGC
GCACCAACCACACGGGGCTCAT|CCGTCAGCCGAAATAGTTA

>874-R1
AGCGATTTCGCCGAGCGGCGTGTGGAAGGGATGTCATCCGTCAGCCGAAATAGTTACACAGGCAGTGCGCTTCCGCGCACCAAC
CACACGGGGCTCAT|CCGTCAGCCGAAATAGTTACACAGGCA

>R1-865
GGATGTCATCCGTCAGCCGAAATAGTTACACAGGCAGTGCGCTTCCGCGCACCAACCACACGGGGCTCAT|CCGTCAGCCGAAATAG
TTACACAGGCAGTGCGCTTCCGCGCACCAACCACACGGG

>R2-865

AGCGATTTCGCCGAGCGGCGTGTGGAAGGGATGTCATCCGTCAGCCGAAATAGTTACACAGGCAGTGCGCTTCCGCGCACCAACCAC
ACGGGGCTCAT|CCGTCAGCCGAAATAGTTACACAGGCA

>R1-875
GCTAAGAAGCGATTTCGCCGAGCGGCGTGTGGCGGGGATGTCATCCGTCAGCCGAAATAGTTACACAGGCAGTGCGCTTCCGCGCAC
CAACCACACGGGGCTCAT|CCGTCAGCCGAAATAGTTAC

>R2-875
GGCCTGTTTCCTAGGCTACAAACGAGGGACTAGTTCCTTATTTACGCCTAGGGGAAAGTCCCCGGACCTAAGGCAGAGAGTGCCACG
TGCGCCTGCACGTAGACTGTCCCTGCACCTCACGGTTA

>the single exon

GCTCGTGCTGAAGGCCTGTTTCCTAGGCTACAAACGAGGGACTAGTTCCTTATTTACGCCTAGGGGAAAGTCCCCGGACCTAAGGCA
GAGAGTGCCACGTGCGCCTGCACGTAGACTGTCCCTGCACCTCACGGTTAATCCGCTAAGAAGCGATTTCGCCGAGCGGCGTGTGGC
AGGGATGTCATCCGTCAGCCGAAATAGTTACACAGGCAGTGCGCTTCCGCGCACCAACCACACGGGGCTCATTCTCAGCACGGC

We are not able to validate the hypothesis of a small circRNA. We cannot exclude that this locus was included in the porcine genome reference sequence with imperfections.

A5 circRNA(s) from the monoexonic gene ENSSSCG00000019194 (*SNORD104)*

To understand the characterization of very small circRNAs, we examined CCRs and their mate pairs selected in the region of *SNORD104*, which is a mono-exonic nc gene. A first LAC was defined by 70 CCRs from Read-1 set and a second was defined by 84 CCRs from Read-2 set. These 70 and 84 CCR are originating from only 86 pairs end (PE). The unbalanced contribution of reads-1 and reads-2 among the list of CCRs is only apparent. The analysis of the sequence of these 86 X2 reads are perfectly compatible with a circular transcript of 71 nt. Nevertheless, the STAR mapping retained CCR including only two segments and it is logical to observe only CCRs of 100 bp while the dataset included 3/4 reads of 125bp.

SNORD104 (Pig Gene, Strain: reference)
ENSSSCG00000019194 12:14867961-14868029:-1

>ref
tggcca GCGGTGATGACATTCCAATAAAGCACGTGTTAGACTGCTGACGCGGGTGATGCGAACAGGAATCTGAGC ccggcc

70 CCRs (all R1) define a first LAC [12:14867960-14868030-] and 84 CCR (all Reads-2) allow define a second LAC [12:14867963-14868033-]). We have only 86 distinct PE and all reads contain the back junction (green|yellow). We did not find any read discordant with this suggested circRNA. The back-junction was identified always once a time per read and only CCR of 100 bp have been retained.

The back junction contains two bases originating from outside of the unique exon described as containing 69 bp. Nevertheless, the transcript of *SNORD104* includes 70 bp in human, bovin, mouse, and rabbit.

Consensus

AAGCACGTGTTAGACTGCTGACGCGGGTGATGCGAACAGGAATCTGAGCC|AGCGGTGATGACATTCCAATAAGCACGT
GTTAGACTGCTGACGCGGGTGATGCGAACAGGAATCTGAGCC|AGCGGTGATGACATTCCAATAAAGCACGTGTTAGAC
T

Sequence suggested for the circRNA

GTGTTAGACTGCTGACGCGGGTGATGCGAACAGGAATCTGAGCC|AGCGGTGATGACATTCCAATAAGCA

Two examples of PE

>1R1
TAAAGCACGTGTTAGACTGCTGACGCGGGTGATGCGAACAGGAATCTGAGCCAGCGGTGATGACATTCCAATAAAGCAC
GTGTTAGACTGCTGACGCGGG

>1R2
TGACATTCCAATAAAGCACGTGTTAGACTGCTGACGCGGGTGATGCGAACAGGAATCTGAGCCAGCGGTGATGACATTC
CAATAAAGCACGTGTTAGACT

>5R1
CACGTGTTAGACTGCTGACGCGGGTGATGCGAACAGGAATCTGAGCCAGCGGTGATGACATTCCAATAAAGCACGTGTT
AGACTGCTGACGCGGGTGATG

>5R2
TGACATTCCAATAAAGCACGTGTTAGACTGCTGACGCGGGTGATGCGAACAGGAATCTGAGCCAGCGGTGATGACATTC
CAATAAAGCACGTGTTAGACT

When the CCR is a read-1, STAR proposes a mapping in considering “GCC|” as the end of the first segment (green) and “|AGC” as the start of the second segment (yellow).

When the CCR is a read-1, STAR proposes a mapping in considering the end of the first segment (yellow) “|GGT”.

The circRNA originating from the unique exon of *SNORD104* is an exonic circRNA

---

set of LACs (size)	CircRNA	N. CCR	chr	position	Size exon	gene_id		gene_biotype	
1x 110 nt	Exonic circRNA	9	**7**	**77,77**	110 bp	ENSSSCG00000030175	7:77770851-77770960+	snoRNA	SNORD8
1x 130 nt	Exonic circRNA	29	**9**	**0,71**	130 bp	ENSSSCG00000039963	9:710016-710145-	snoRNA	novel gene
1x 145 nt	Exonic circRNA	23	**9**	**9,629**	145 bp	ENSSSCG00000025715	9:9629462-9629606+	snoRNA	SNORD15
1x 187 nt	Probable exonic circRNA	112	**13**	**75,694**	187 bp	(1)	13:75694365-75694550+		novel nc-SSC13:75Mb
1x 252 nt	Probable exonic circRNA	63	**1**	**87,223**	250 bp	(2)	1:87223423-87223674+		novel nc-SSC1:87Mb
2x 71 nt	Probable exonic circRNA	154	**12**	**14,867**	69 bp*	ENSSSCG00000019194	12:14867961-14868029-	snoRNA (RNA modification guide)	SNORD104
2x 73 nt	Sub-exonic circRNAs	26	**6**	**54,567**	81 bp	ENSSSCG00000025540	6:54567160-54567240+	snoRNA (RNA modification guide)	SNORD33
1 x 99 nt + 2 x 53nt	Sub-exonic circRNAs	15 + 37	**9**	**109,291**	102 bp	ENSSSCG00000018101	9:109291395-109291496-	(misc_RNA)**	Y_RNA
1x 100 nt	Sub-exonic circRNAs	5	**2**	**77,49**	107 bp	ENSSSCG00000028716	2:77490589-77490695-	Spliceosomal RNA	U6
1x 97 nt	Sub-exonic circRNAs	9	**9**	**109,287**	113 bp	ENSSSCG00000019375	9:109287974-109288086+	(misc_RNA)**	Y_RNA
1x 114 nt	Sub-exonic circRNAs	7	**7**	**85,594**	133 bp	ENSSSCG00000030477	6:85594569-85594701-	snoRNA (RNA modification guide)	SNORA61
1x 88 nt	Sub-exonic circRNAs	6	**18**	**50,374**	134 bp	ENSSSCG00000021904	18:50374218-50374351+	snoRNA (RNA modification guide)	SNORA5A
15x 107-124 nt	Sub-exonic circRNAs	503	**12**	**52,87**	135 bp	ENSSSCG00000018563	12:52870367-52870501+	snoRNA (RNA modification guide)	SNORA48
2x 134-136 nt	Sub-exonic circRNAs	21	**9**	**117,939**	136 bp	ENSSSCG00000034100	9:117939334-117939469-	scaRNA (RNA modification guide)	SCARNA3
2x 126 nt	Sub-exonic circRNAs	27	**15**	**120,3**	138 bp	ENSSSCG00000037505	15:120300270-120300407+	snoRNA (RNA modification guide)	novel gene
8x 93-138 nt	Sub-exonic circRNAs	165	**2**	**48,674**	141 bp	ENSSSCG00000019944	2:48674192-48674332+	snoRNA (RNA modification guide)	SNORD97
1x 139 nt	Sub-exonic circRNAs	26	**12**	**52,872**	141 bp	ENSSSCG00000019619	12:52872048-52872188+	snoRNA (RNA modification guide)	SNORD10
9x 71-193 nt	Sub-exonic circRNAs	79	**12**	**34,885**	216 bp	ENSSSCG00000040361	12:34885727-34885942-	Spliceosomal RNA	U3
38x 91-199 nt +7 x 60-63 nt	Sub-exonic circRNAs	1750 + 224	**1**	**263,38**	258 bp	ENSSSCG00000018700	1:236380019-236380276-	ribozyme (catalytic RNAs)	RNase_MRP
1x 204 nt	Sub-exonic circRNAs	17	**1**	**179,686**	300 bp	ENSSSCG00000031740	1:179686395-179686694-	misc_RNA	Metazoa_SRP
9x 134-271 nt	Sub-exonic circRNAs	294	**7**	**78,551**	327 bp	ENSSSCG00000020439	7:78551527-78551853+	ribozyme (catalytic RNAs)	RNaseP_nuc
7x 76-144 nt	Sub-exonic circRNAs	83	**5**	**64,155**	329 bp	ENSSSCG00000040520	5:64155664-64155992-	scaRNA (RNA modification guide)	SCARNA10
5x 350-358 nt	Sub-exonic circRNAs	122	**1**	**0,065**	350/360 bp	(3) seq in Suppl. Doc. 3C	1:65221-65580-		novel nc-SSC1:65kb
20x 88-244 nt	Sub-exonic circRNAs	1016	**7**	**10,867**	515 bp	(4) seq in Suppl. Doc. 3C	7:10867825-10868339-	probable ribosomal	novel nc-SSC7:10Mb
5x 102-118 nt	Sub-exonic circRNAs	53	**16**	**37,934**	125 bp	(5) seq in Suppl. Doc. 3C	16:37934981-37935105-	probable ribosomal	novel nc-SSC16:37Mb
2x 268-271 nt	Probable lariat-derived intronic circRNA	55	**1**	**269,077**		(6)	1:269077432-269077702+	annotation of *PKN3* not OK	novel nc-SSC1:269Mb
3x 78-92 nt	Sub-exonic circRNAs	32	**7**	**20,775**	640 bp	ENSSSCG00000033398		coding-gene	ENSSSCG00000033398 ***
1x 123 nt	Sub-exonic circRNAs	5	**8**	**67,161**	1203 bp	ENSSSCG00000039238		coding-gene	CABS1

Suppl. Doc. 3B **CircRNAs and mono-exonic genes** **Testis-31**

These six loci are highlighted after the manual/visual examination of the list of LACs

(1) possible novel exon of a new monoexonic gene (186 pb); circRNAs located at the 5'end of an intron (1,385bp) of *CEP63* gene (ENSSSCG00000011645)

(2) possible novel exon of a new monoexonic gene (251 pb); circRNAs located at the 3'end of an intron (2,711 bp) of PHIP gene (ENSSSCG00000004473)

(3) possible novel exon of a new monoexonic gene (350 pb); circRNAs located in intergenic region

(4) possible novel exon of a new monoexonic gene (515 pb); circRNAs located in intergenic region

(5) possible novel exon of a new monoexonic gene (125 pb); circRNA located in an intron (36,418 bp) of PDE4D gene (ENSSSCG00000016929)

(6) possible lariat-derived intronic circRNA in a improper annotated gene (*PKN3* - ENSSSCG00000005657)

* see Suppl. Doc. 2A2

** release 97 => misc_RNA release 98 => Y RNA

*** This gene has been retired of the release 98

Supplementary Document 3B

>novel nc-SSC16:37Mb

AGCAGTTGCACCATTTGGGTGTCCTGATCCAACATCGAGGTCGTAAACCCTATTGTCGATAGGAACTC
TAGAATAGGATTGCGCTGTTATCCCTAGGGTAACTTGTTCCGTTGATCAAAATTTTG

>novel nc-SSC1:65kb

GGTGACAGGACCTCCATCAGCGTGGGTCCCGAGCAACCCCGTGGACCAGGGAGCCCCAGCACTGCTGC
AACCATGCCTGGGAACACGTACCAGGAACTTCTCAGGAATGAGAAGGAACCTTCTGCTGGGTGGGCCA
CTCAGACTTCAGGTCACGTTTGCCACAGCAGCTGGTGCCACCTTCCCACCTCAGCAGAGTCTCCTTTG
CTTGGGGGTTAACAGTAGGACTTGTTCCCGAAGCTGTACCTCCAGCAGCTGCTGGCCCTTAGCCAGAA
GCATGACACGCCGTCACCTGCTTCTGCTGTCTCAAGCCCCCGGGACCTGAGCCCTGGGCGGGGCGCTG
GGGTTGCTCC
>novel nc-SSC7:10Mb

CTGATCGTTTTTTCACTGACCCGGTGAGGCGGGGAGGCGAGCCCCGAGGGGCTCTCGCTTCTGGCGCC
AAGCGCCCGGCCGCGCGCCGGCCGGGCGCGACCCGCTCCGGGGACAGTGCCAGGTGGGGAGTTTGACT
GGGGCGGTACACCTGTCAAACGGTAACGCAGGTGTCCTAAGGCGAGCTCAGGGAGGACAGAAACCTCC
CGTGGAGCAGAAGGGCAAAAGCTCGCTTGATCTTGATTTTCAGTACGAATACAGACCGTGAAAGCGGG
GCCTCACGATCCTTCTGACCTTTGGGGTTTTAAGCAGGAGGTGTCAGAAAAGTTACCACAGGATAAC
TGGCTTGTGGCGGCCAAGCGTTCATAGCGACGTCGCTTTTTGATCCTTCGATGTCGGCTCTTCCTATC
ATTGTGAAGCAGAATTCACCAAGCGTTGGATTGTTCACCCACTAATAGGGAACGTGAGCTGGGTTTAG
ACCGTCGTGAGACAGGTTAGTTTTACCCTACTGATGATG
